# Exploring mixture estimators in stratified random sampling

**DOI:** 10.1371/journal.pone.0307607

**Published:** 2024-09-17

**Authors:** Kanwal Iqbal, Syed Muhammad Muslim Raza, Tahir Mahmood, Muhammad Riaz

**Affiliations:** 1 Department of Economics and Statistics, Dr Hasan Murad School of Management (HSM), University of Management and Technology, Lahore, Pakistan; 2 Department of Mathematics and Statistics, University of Lahore, Sargodha Campus, Sargodha, Pakistan; 3 Department of Statistics, Virtual University of Pakistan, Lahore, Pakistan; 4 School of Computing, Engineering and Physical Sciences, University of the West of Scotland, Paisley, United Kingdom; 5 Department of Mathematics, College of Computing and Mathematics, King Fahd University of Petroleum and Minerals, Dhahran, Saudi Arabia; Cairo University, EGYPT

## Abstract

Advancements in sensor technology have brought a revolution in data generation. Therefore, the study variable and several linearly related auxiliary variables are recorded due to cost-effectiveness and ease of recording. These auxiliary variables are commonly observed as quantitative and qualitative (attributes) variables and are jointly used to estimate the study variable’s population mean using a mixture estimator. For this purpose, this work proposes a family of generalized mixture estimators under stratified sampling to increase efficiency under symmetrical and asymmetrical distributions and study the estimator’s behavior for different sample sizes for its convergence to the Normal distribution. It is found that the proposed estimator estimates the population mean of the study variable with more precision than the competitor estimators under Normal, Uniform, Weibull, and Gamma distributions. It is also revealed that the proposed estimator follows the Cauchy distribution when the sample size is less than 35; otherwise, it converges to normality. Furthermore, the implementation of two real-life datasets related to the health and finance sectors is also presented to support the proposed estimator’s significance.

## Introduction

Sampling is a procedure of selecting a representative fraction of a population so that one may observe and estimate something about the characteristics of interest for the entire population [1, 2]. A primary objective of survey sampling is to achieve a practical design for the survey to attain an adequate sample size for estimating the parameters of interest for the population under study. Survey sampling has several advantages over a full population study, including lower resource consumption, shorter turnaround times, and lower costs [3]. Moreover, it also provides a basis for acquiring precise and useful parameter estimations.

Additionally, survey sampling makes generating accurate and efficient parameter estimates easier. Survey statisticians can improve an estimator’s efficiency by enhancing the sampling technique, boosting the sample size, or employing auxiliary data. Auxiliary information about the population can consist of a known variable to which the study variable is approximately related. Typically, this auxiliary information is easy to quantify, whereas measuring the study variable itself can be costly [4]. By using this additional data, which includes characteristics and variables that are highly correlated with the variable of interest, the estimation process can improve the accuracy of the study variable’s mean [5, 6]. While measuring the study variable can often be expensive, the auxiliary information is usually easy to quantify. The *i*^th^ element of *Y* may be correlated with one or more auxiliary variables (*X*_*i*_) in addition to the study variable *Y*. The average elevation, area, and type of vegetation in a cattle field are examples of auxiliary variables that may be included if the study variable is the number of animals in the field. Auxiliary data is used in survey sampling for three main purposes: pre-selection, selection (i.e., selecting units that correspond to the study variable based on the auxiliary variable), and estimation (i.e., creating estimators of the ratio, product, and regression type).

In many studies, survey statisticians have been keenly interested in estimating parameters for the heterogeneous population. Therefore, for the heterogeneous population, Neyman [7] introduced Stratified Random Sampling (StRS), in which the population is partitioned into groups called "strata." Then a sample is chosen by some pattern within each stratum, and independent selections are made in different groups. Stratification is a probability sampling design used to increase the precision of estimation [8]. StRS’s sampling methodology ensures that every demographic group is adequately represented in the sample. For small sample sizes, adequate precision and accuracy may be achieved by using StRS’s procedure. In sample selection, the StRS design helps to minimize bias. From an organizational point of view, stratified sampling is very convenient. Moreover, it is also recommended that auxiliary information be used in the StRS population parameter estimation process, which is useful for comparing estimates among several population groups. For more recent studies on StRS, see studies [9–11] and references therein.

Historically, numerous researchers have independently utilized auxiliary variables and attributes, proposing diverse estimators within the StRS framework to enhance efficiency [12]. Cochran [13] developed classical ratio and regression methods to calculate the study variable’s population mean. Graunt [14] was the first known user of the ratio estimator. When estimating using a ratio, Cochran first used auxiliary information. To calculate the population mean, Robson [15] suggested product type estimators by incorporating the ancillary data. Kadilar and Cingi [16] proposed the estimator when the population coefficient of skewness and kurtosis are unknown in stratified random sampling. In the estimation of population mean three cases of using auxiliary information have been suggested by Samiuddin and Hanif [17], such as no information, full information, and partial information. Ahmad, Hanif [18] suggested a modified and efficient estimator of the population mean using two auxiliary variables in survey sampling. Ahmad, Hanif [19] established a generalized multi-phase multivariate regression estimator with the help of several auxiliary variables. To find the population mean, Moeen, Shahbaz [20] suggested mixture estimators by simultaneously utilizing auxiliary variables and attributes. Double-phase and multi-phase sampling of a vector of variables of interest are used to calculate the population mean. Malik and Singh [21] also worked on the stratified sampling estimator. In single-phase sampling, Verma, Sharma [22] suggested some modified regression-cum-ratio, and exponential ratio type estimators. The improved version of the exponential estimator of the mean under StRS, initially proposed by Zaman [23], is presented by Singh, Ragen [24], and its properties for big sample approximation. Singh, Ragen [24] also suggested a class of estimators of population variance along with their asymptotic properties. Zaman [25] established a class of ratio-type estimators with the help of auxiliary attributes to calculate the population’s mean. Under the StRS design, mixture regression cum ratio estimators in a single-phase scheme were established by Moeen [26]. Yadav and Zaman [27] suggested a class of estimators using both conventional and non-conventional auxiliary variable parameters. Under StRS design, using an auxiliary attribute, Zaman and Kadilar [28] suggested exponential ratio estimators for stratified two-phase sampling. Lawson and Thongsak [29] introduce a novel set of population mean estimators designed for use in stratified random sampling. Their study examines the bias and mean square error of these estimators using Taylor series approximation. Through simulation and application to air pollution data in northern Thailand, they evaluate the performance of the estimators. Results from the air pollution data show that the proposed estimators outperform others in terms of efficiency.

Many sampling survey investigations seek to devise an efficient estimator for the population mean. Numerous studies have been designed to pursue this aim, incorporating various adaptations to classical ratio, product, and regression estimators utilizing SRS and StRS. Kadilar and Cingi [16] and Zaman [25] employed auxiliary variables and attributes, respectively, proposing different estimators within the StRS framework. However, these estimators prove beneficial only in specific scenarios, such as when auxiliary variables or attributes are solely utilized to estimate the population mean of the study variable. Consequently, a gap exists in the literature concerning the simultaneous utilization of auxiliary attributes and auxiliary variables alongside the study variable to estimate the population parameter. For example, in a household survey, income, expenditures, family size, number of employed, and number of literate persons are related variables that can be used as auxiliary information for estimating any characteristic. So, from the above example, we can use income (a quantitative variable) and family size (a qualitative variable) simultaneously to estimate the expenditure (a study variable). Therefore, the current article aims to propose a class of generalized mixture ratio estimators to estimate the population mean of the study variable by simultaneously incorporating the auxiliary attributes (qualitative) and variables (quantitative) in stratified random sampling (StRS). Therefore, the suggested estimators could be used in various sampling surveys.

The subsequent sections of the paper are organized as follows: Section: “Notations under Stratified Random Sampling” offers an overview of stratified random sampling. Section: “A Family of Proposed Estimators under Stratified Random Sampling” introduces the proposed estimators. In Section: “A Simulation Study”, a comparative analysis is conducted based on the simulation study. Section: “Illustrative Examples” showcases two real-life examples. Finally, Section: “Conclusion and Recommendations” outlines the conclusions drawn from the study and provides recommendations for future research.

## Notations under stratified random sampling

Let *N* denote the size of the population, *N* = *N*_1_ + *N*_2_ + *N*_3_ +…‥ + *N*_*h*_+.… + *N*_*k*_, where the number of units in stratum *h* is represented by *N*_*h*_, *n* = *n*_1_ + *n*_2_ + *n*_3_+…‥ +*n*_*h*_ +….+*n*_*k*_, where the number of sampling units in stratum *h* is represented by *n*_*h*_, *Y* stands for the study variable, *X* refers to the auxiliary variable, *P* is the population proportion auxiliary attribute, and *X* and *Y* have a strong correlation. The ${\bar{Y}}_{h}$ refers to the population mean of the variable of interest *Y* in stratum *h*, ${\bar{X}}_{h}$ is the population mean of auxiliary variable *X* in stratum *h*, *P*_*h*_ is the population proportion in auxiliary attribute in stratum *h*. ${\overset{⌢}{\bar{X}}}_{h}$ and ${\bar{Y}}_{h}$ are the sample means of *Y* and *X* in stratum *h*. ${\hat{P}}_{h}$ is the sample proportion in auxiliary attribute in stratum *h* $W_{h}=\frac{N_{h}}{N},W_{h}^{2}=\frac{N_{h}^{2}}{N^{2}}$ is the stratum weight. The $S_{yh}^{2}$ and $S_{xh}^{2}$ are the variances of stratum *h*, *C*_*yh*_ and *C*_*xh*_ are the coefficients of variations in stratum *h*. $\rho_{xy_{h}}$ is the correlation coefficient between *X* and *Y* in stratum *h*, *β*_2(*xh*)_ and *β*_1(*xh*)_ are the kurtosis and skewness in stratum *h* and $C_{xyh}=\rho_{xyh}C_{xh}C_{yh}$ Let′s say that an attribute j = 1,2,3,…,m is dichotomized in the population based on its presence or absence; the attribute’s values should be "0" and "1" correspondingly.


$$\tau_{ij_{h}}=\left\{\begin{array}{c} 1,\\ 0, \end{array}\begin{array}{c} \;\text{if}\;h^{th}\;\text{unit}\;\text{of}\;\text{the}\;\text{population}\;\text{possesses}\;\text{attribute}\\ \text{otherwise} \end{array}\right.$$

Under stratified random sampling, we take into account the following notations in order to determine the biases and mean square error (MSE) of the suggested estimator: $e_{0}=\frac{{\bar{y}}_{h}-{\bar{Y}}_{h}}{{\bar{Y}}_{h}},{\bar{y}}_{h}={\bar{Y}}_{h}\left(1+e_{0}\right),e_{3}=\frac{p_{U_{h}}-P_{U_{h}}}{P_{U_{h}}},p_{U_{h}}=P_{U_{h}}\left(1+e_{3}\right),e_{4}=\frac{{\bar{x}}_{U_{h}}-{\bar{x}}_{U_{h}}}{{\bar{X}}_{U_{h}}},{\bar{x}}_{U_{h}}={\bar{X}}_{U_{h}}(1+e_{4}),$ $E\left(e_{0}\right)=E\left(e_{3}\right)=E\left(e_{4}\right)=0,E\left(e_{0}^{2}\right)=\frac{\sum_{h=1}^{k}\omega_{h}^{2}\lambda_{h}S_{yh}^{2}}{{\bar{Y}}_{h}^{2}},E\left(e_{3}^{2}\right)=\frac{\sum_{h=1}^{k}\omega_{h}^{2}\lambda_{h}S_{ph}^{2}}{P_{Uh}^{2}},E\left(e_{4}^{2}\right)=\frac{\sum_{h=1}^{k}\omega_{h}^{2}\lambda_{h}S_{xh}^{2}}{{\bar{X}}_{Uh}^{2}},$ $E\left(e_{0}e_{3}\right)=\frac{\sum_{h=1}^{k}\omega_{h}^{2}\lambda_{h}S_{yph}}{{\bar{Y}}_{h}\cdot P_{U_{h}}},E\left(e_{0}e_{4}\right)=\frac{\sum_{h=1}^{k}\omega_{h}^{2}\lambda_{h}S_{yxh}}{{\bar{Y}}_{h}\cdot {\bar{X}}_{U_{h}}},E\left(e_{3}e_{4}\right)=\frac{\sum_{h=1}^{k}\omega_{h}^{2}\lambda_{h}s_{xph}}{P_{U_{h}}{\bar{X}}_{U_{h}}},\text{and}\;\lambda_{h}=\left\{\frac{1}{n_{h}}-\frac{1}{N_{h}}\right\}.$

### Relevant estimators under StRS

This section discusses some existing estimators and their bias and mean square error (MSE). Kadilar and Cingi [16] proposed the following estimators:

$${\hat{\mu }}_{yKS_{1},StRS}={\bar{y}}_{st}\frac{\sum_{h=1}^{k}\omega_{h}\left({\bar{X}}_{h}+C_{xh}\right)}{\sum_{h=1}^{k}\omega_{h}\left({\hat{\bar{X}}}_{h}+C_{xh}\right)}.$$ (1)

$$MSE({\hat{\mu }}_{yKS_{1},StRS})=\sum_{h=1}^{k}\omega_{h}^{2}\lambda_{h}[S_{yh}^{2}-2RS_{syxh}+R^{2}S_{xh}^{2}].$$ (2)

$${\hat{\mu }}_{yKS_{2},StRS}={\bar{y}}_{st}\frac{\sum_{h=1}^{k}\omega_{h}\left({\bar{X}}_{h}+\beta_{(2h)x}\right)}{\sum_{h=1}^{k}\omega_{h}\left({\hat{\bar{X}}}_{h}+\beta_{(2h)x}\right)}.$$ (3)

$$MSE({\hat{\mu }}_{yKS_{2},StRS})=\sum_{h=1}^{k}\omega_{h}^{2}\lambda_{h}[S_{yh}^{2}-2RS_{syxh}+R^{2}S_{xh}^{2}].$$ (4)

$${\hat{\mu }}_{yKS_{3},StRS}={\bar{y}}_{st}\frac{\sum_{h=1}^{k}\omega_{h}\left({\bar{X}}_{h}C_{xh}+\beta_{(2h)x}\right)}{\sum_{h=1}^{k}\omega_{h}\left({\hat{\bar{X}}}_{h}C_{xh}+\beta_{(2h)x}\right)}.$$ (5)

$$MSE({\hat{\mu }}_{yKS_{3},StRS})=\sum_{h=1}^{k}\omega_{h}^{2}\lambda_{h}[S_{yh}^{2}-2RS_{syxh}+R^{2}S_{xh}^{2}].$$ (6)

$${\hat{\mu }}_{yKS_{4},StRS}={\bar{y}}_{st}\frac{\sum_{h=1}^{k}\omega_{h}\left({\bar{X}}_{h}\beta_{(2h)x}+C_{xh}\right)}{\sum_{h=1}^{k}\omega_{h}\left({\hat{\bar{X}}}_{h}\beta_{(2h)x}+C_{xh}\right)}.$$ (7)

$$MSE({\hat{\mu }}_{yKS_{4},StRS})=\sum_{h=1}^{k}\omega_{h}^{2}\lambda_{h}[S_{yh}^{2}-2RS_{syxh}+R^{2}S_{xh}^{2}].$$ (8)

In stratified random sampling by using the auxiliary attribute, Zaman [25] proposed the following estimators:

$${\hat{\mu }}_{yZ_{1},StRS}={\bar{y}}_{st}\frac{\sum_{h=1}^{k}\omega_{h}\left(P_{h}+C_{ph}\right)}{\sum_{h=1}^{k}\omega_{h}\left({\hat{P}}_{h}+C_{ph}\right)}.$$ (9)

$$MSE({\hat{\mu }}_{yZ_{1},StRS})=\sum_{h=1}^{k}\omega_{h}^{2}\lambda_{h}[S_{yh}^{2}-2RS_{syph}+R^{2}S_{ph}^{2}].$$ (10)

$${\hat{\mu }}_{yZ_{2},StRS}={\bar{y}}_{st}\frac{\sum_{h=1}^{k}\omega_{h}\left(P_{h}+\beta_{2h(\vartheta )}\right)}{\sum_{h=1}^{k}\omega_{h}\left({\hat{P}}_{h}+\beta_{2h(\vartheta )}\right)}.$$ (11)

$$MSE({\hat{\mu }}_{yZ_{2},StRS})=\sum_{h=1}^{k}\omega_{h}^{2}\lambda_{h}[S_{yh}^{2}-2RS_{syph}+R^{2}S_{ph}^{2}].$$ (12)

$${\hat{\mu }}_{yZ_{3},StRS}={\bar{y}}_{st}\frac{\sum_{h=1}^{k}\omega_{h}\left(P_{h}+\rho_{yph}\right)}{\sum_{h=1}^{k}\omega_{h}\left({\hat{P}}_{h}+\rho_{yph}\right)}.$$ (13)

$$MSE({\hat{\mu }}_{yZ_{3},StRS})=\sum_{h=1}^{k}\omega_{h}^{2}\lambda_{h}[S_{yh}^{2}-2RS_{syph}+R^{2}S_{ph}^{2}].$$ (14)

$${\hat{\mu }}_{yZ_{4},StRS}={\bar{y}}_{st}\frac{\sum_{h=1}^{k}\omega_{h}\left(P_{h}C_{ph}+\beta_{2h(\vartheta )}\right)}{\sum_{h=1}^{k}\omega_{h}\left({\hat{P}}_{h}C_{ph}+\beta_{2h(\vartheta )}\right)}.$$ (15)

$$MSE({\hat{\mu }}_{yZ_{4},StRS})=\sum_{h=1}^{k}\omega_{h}^{2}\lambda_{h}[S_{yh}^{2}-2RS_{syph}+R^{2}S_{ph}^{2}].$$ (16)

$${\hat{\mu }}_{yZ_{5},StRS}={\bar{y}}_{st}\frac{\sum_{h=1}^{k}\omega_{h}\left(P_{h}\beta_{2h(\vartheta )}+C_{ph}\right)}{\sum_{h=1}^{k}\omega_{h}\left({\hat{P}}_{h}\beta_{2h(\vartheta )}+C_{ph}\right)}.$$ (17)

$$MSE({\hat{\mu }}_{yZ_{5},StRS})=\sum_{h=1}^{k}\omega_{h}^{2}\lambda_{h}[S_{yh}^{2}-2RS_{syph}+R^{2}S_{ph}^{2}].$$ (18)

### A family of proposed estimators under stratified random sampling

Within this section, we introduce a novel class of generalized mixture estimator, building upon the framework proposed by Zaman [25]; this estimator will be suitable for the estimation of the population mean $\bar{Y}$, incorporating the concurrent utilization of auxiliary variables and attributes within a stratified random sampling context.

$$T_{KMst}=\sum_{h=1}^{k}\frac{N_{h}}{N}\left[aT_{p_{h}}+(1-a)T_{x_{h}}\right],$$ (19)

where

$$T_{p_{h}}={\bar{y}}_{h}P_{U},\;T_{x_{h}}={\bar{y}}_{h}{\bar{X}}_{U},$$ (20)

$$\left.\begin{array}{c} P_{U}=\left(P_{h}K_{1}+K_{2}\right)\;\text{and}\;{\bar{X}}_{U}=\left({\bar{X}}_{h}K_{3}+K_{4}\right)\\ p_{U}=\left({\hat{P}}_{h}K_{1}+K_{2}\right)\;\text{and}\;{\hat{\bar{X}}}_{U}=\left({\hat{\bar{X}}}_{h}K_{3}+K_{4}\right) \end{array}\right\}.$$ (21)

Given that where *a* and K are constant, with *a* taking on the values 0 and 1 and *K* ∈ ℝ. Consequently, *K*_1_,*K*_2_,*K*_3_,and *K*_4_ may consist of any real number. Eq (19) can be rewritten as follows using Eq (20):

$$T_{KMst}=\sum_{h=1}^{k}\frac{N_{h}}{N}\left[a\left\{{\bar{y}}_{h}\frac{P_{U}}{{\hat{P}}_{U}}\right\}+(1-a)\left\{{\bar{y}}_{h}\frac{{\bar{X}}_{U}}{{\hat{\bar{X}}}_{U}}\right\}\right].$$ (22)

However, for the *h*^*th*^stratum, let $T_{KM_{h}}$ be a mixture estimator of the population mean given as follows:

$$T_{KM_{h}}=\left[a\left\{{\bar{y}}_{h}\frac{P_{U}}{{\hat{P}}_{U}}\right\}+(1-a)\left\{{\bar{y}}_{h}\frac{{\bar{X}}_{U}}{{\hat{\bar{X}}}_{U}}\right\}\right].$$ (23)

### Derivation of $Bias\left(T_{KM_{h}}\right)$ and $MSE\left(T_{KM_{h}}\right)$

Similarly, we can rewrite Eq (23) as follows by using ${\bar{y}}_{h}$ as a common factor:

$$T_{KM_{h}}={\bar{y}}_{h}\left[a\left\{\frac{P_{U}}{{\hat{P}}_{U}}\right\}+(1-a)\left\{\frac{{\bar{X}}_{U}}{{\hat{\bar{X}}}_{U}}\right\}\right],$$ (24)

Using notations from preceding section, the bias of $T_{KM_{h}}$ is derived as follows, and after simplification, we obtain,

$$T_{KM_{h}}={\bar{Y}}_{h}\left(1+e_{0}\right)\left[a\left\{\frac{P_{U_{h}}}{{\hat{P}}_{U_{h}}\left(1+e_{3}\right)}\right\}+(1-a)\left\{\frac{{\bar{X}}_{U_{h}}}{{\hat{\bar{X}}}_{U_{h}}\left(1+e_{4}\right)}\right\}\right].$$

Similarly, using notations from preceding section and after simplification, we get,

$$Bias[T_{KM_{h}}]=\left(\frac{{\bar{Y}}_{h}S_{x_{h}}^{2}}{{\bar{X}}_{U_{h}}^{2}}-\frac{S_{y_{h}x_{h}}}{{\bar{X}}_{U_{h}}}\right)+a\left(\frac{{\bar{Y}}_{h}S_{p_{h}}^{2}}{P_{U_{h}}^{2}}-\frac{S_{y_{h}p_{h}}}{P_{U_{h}}}-\frac{{\bar{Y}}_{h}S_{x_{h}}^{2}}{{\bar{X}}_{U_{h}}^{2}}+\frac{S_{y_{h}x_{h}}}{{\bar{X}}_{U_{h}}}\right).$$ (25)

Hence, the Bias expression of $T_{KM_{st}}$ is obtained as follows:

$$Bias\left(T_{KMst}\right)=\sum_{h=1}^{k}\frac{N_{h}^{2}}{N^{2}}Bias\left[T_{KM_{h}}\right]$$

$$Bias\left(T_{KMst}\right)=\sum_{h=1}^{k}\frac{N_{h}^{2}}{N^{2}}\left(\frac{{\bar{Y}}_{h}S_{x_{h}}^{2}}{{\bar{X}}_{U_{h}}^{2}}-\frac{S_{y_{h}x_{h}}}{{\bar{X}}_{U_{h}}}\right)+a\sum_{h=1}^{k}\frac{N_{h}^{2}}{N^{2}}\left(\frac{{\bar{Y}}_{h}S_{p_{h}}^{2}}{P_{U_{h}}^{2}}-\frac{S_{y_{h}p_{h}}}{P_{U_{h}}}-\frac{{\bar{Y}}_{h}S_{x_{h}}^{2}}{{\bar{X}}_{U_{h}}^{2}}+\frac{S_{y_{h}x_{h}}}{{\bar{X}}_{U_{h}}}\right).$$ (26)

and

$$MSE\left(T_{KM_{h}}\right)=\left[\begin{array}{c} \lambda_{h}\left(S_{y_{h}}^{2}+\frac{{\bar{Y}}_{h}^{2}S_{x_{h}}^{2}}{{\bar{X}}_{U_{h}}^{2}}-\frac{2{\bar{Y}}_{h}S_{yx_{h}}}{{\bar{X}}_{U_{h}}}\right)+a^{2}\lambda_{h}\left(\frac{{\bar{Y}}_{h}^{2}S_{p}^{2}}{P_{U_{h}}^{2}}+\frac{{\bar{Y}}_{h}^{2}S_{x}^{2}}{{\bar{X}}_{U_{h}}^{2}}-\frac{2{\bar{Y}}_{h}^{2}S_{xp}}{{\bar{X}}_{U_{h}}.P_{U_{h}}}\right)\\ +2a\lambda_{h}\left(\frac{{\bar{Y}}_{h}S_{yx_{h}}}{{\bar{X}}_{U_{h}}}-\frac{{\bar{Y}}_{h}S_{yp_{h}}}{P_{U_{h}}}+\frac{{\bar{Y}}_{h}^{2}S_{xp_{h}}}{{\bar{X}}_{U_{h}}.P_{U_{h}}}-\frac{{\bar{Y}}_{h}^{2}S_{x_{h}}^{2}}{{\bar{X}}_{U_{h}}^{2}}\right) \end{array}\right].$$ (27)

Moreover, the following simplification can be used to determine the maximum value of " *a* ":

$$a=\frac{{\bar{X}}_{U_{h}}.P_{U_{h}}\left[-S_{yx_{h}}P_{U_{h}}+S_{yp_{h}}{\bar{X}}_{U_{h}}\right]+{\bar{Y}}_{h}P_{U_{h}}\left[-S_{xp_{h}}{\bar{X}}_{U_{h}}+S_{x_{h}}^{2}P_{U_{h}}\right]}{{\bar{Y}}_{h}[S_{p_{h}}^{2}{\bar{X}}_{U_{h}}^{2}+S_{x_{h}}^{2}P_{U_{h}}^{2}+2S_{xp_{h}}{\bar{X}}_{U_{h}}.P_{U_{h}}]}.$$ (28)

Adding the value of *a* in Eq (27) and after simplification, we obtained,

$$MSE\left(T_{KM_{h}}\right)=\left[\lambda_{h}\left(S_{y_{h}}^{2}+\frac{{\bar{Y}}_{h}^{2}S_{x_{h}}^{2}}{{\bar{X}}_{U_{h}}^{2}}-\frac{2{\bar{Y}}_{h}S_{yx_{h}}}{{\bar{X}}_{U_{h}}}\right)+\lambda_{h}\frac{\left[-S_{yx_{h}}P_{U_{h}}+S_{yp_{h}}{\bar{X}}_{U_{h}}\right]+\frac{{\bar{Y}}_{h}}{{\bar{X}}_{U_{h}}}{\left[-S_{xp_{h}}{\bar{X}}_{U_{h}}+S_{x_{h}}^{2}P_{U_{h}}\right]}^{2}}{S_{p_{h}}^{2}{\bar{X}}_{U_{h}}^{2}+S_{x_{h}}^{2}P_{U_{h}}^{2}+2S_{xp_{h}}{\bar{X}}_{U_{h}}.P_{U_{h}}}\right].$$ (29)

Hence, the mean square error (MSE) expression of $T_{KM_{st}}$ is obtained as follows:

$$MSE\left(T_{KMst}\right)=\sum_{h=1}^{k}\frac{N_{h}^{2}}{N^{2}}MSE\left(T_{KM_{h}}\right)$$

$$MSE\left(T_{KMst}\right)=\sum_{h=1}^{k}\frac{N_{h}^{2}}{N^{2}}\left(\left(\lambda_{h}\left(S_{y_{h}}^{2}+\frac{{\bar{Y}}_{h}^{2}S_{x_{h}}^{2}}{{\bar{X}}_{U_{h}}^{2}}-\frac{2{\bar{Y}}_{h}S_{yx_{h}}}{{\bar{X}}_{U_{h}}}\right)+\lambda_{h}\frac{\left[-S_{yx_{h}}P_{U_{h}}+S_{yp_{h}}{\bar{X}}_{U_{h}}\right]+\frac{{\bar{Y}}_{h}}{{\bar{X}}_{U_{h}}}{\left[-S_{xp_{h}}{\bar{X}}_{U_{h}}+S_{x_{h}}^{2}P_{U_{h}}\right]}^{2}}{S_{p_{h}}^{2}{\bar{X}}_{U_{h}}^{2}+S_{x_{h}}^{2}P_{U_{h}}^{2}+2S_{xp_{h}}{\bar{X}}_{U_{h}}.P_{U_{h}}}\right)\right).$$ (30)

The complete derivation of $Bias(T_{KM_{h}})$ and $MSE(T_{KM_{h}})$ given in Eqs (25–29) are provided in S1 Appendix.

### Unique cases of the proposed mixture estimator

In this section, we will delve into specific cases of the proposed mixture estimator, examining various combinations of constants. While Tables 1 and 2 highlight certain special cases of the estimator, exploring additional combinations of constants can reveal further special cases not covered in the tables.

**Table 1:** Particular situations for the proposed mixture estimator for *a* = 0.

*K*_1_	*K*_2_	*K*_3_	*K*_4_	Estimators
***β*_2_**(*p*_*h*_)	*C*_*ph*_	*β*_2_(*x*_*h*_)	*β*_1_(*p*_*h*_)	$T_{s_{1}}={\bar{y}}_{h}\sum_{h=1}^{k}W_{h}\left[\frac{{\bar{X}}_{h}\beta_{2\left(x_{h}\right)}+\beta_{1\left(p_{h}\right)}}{{\hat{\bar{X}}}_{h}\beta_{2\left(x_{h}\right)}+\beta_{1\left(p_{h}\right)}}\right]$
***β*_2_**(*p*_*h*_)s	*C*_*ph*_	*β*_2_(*x*_*h*_)	*σ*_*h*_	$T_{s_{2}}={\bar{y}}_{h}\sum_{h=1}^{k}W_{h}\left[\frac{{\bar{X}}_{h}\beta_{2\left(x_{h}\right)}+\sigma_{h}}{{\bar{\hat{X}}}_{h}\beta_{2\left(x_{h}\right)}+\sigma_{h}}\right]$
***β*_2_**(*p*_*h*_)	*C*_*ph*_	*β*_2_(*x*_*h*_)	*ρ*_*h*_	$T_{s_{3}}={\bar{y}}_{h}\sum_{h=1}^{k}W_{h}\left[\frac{{\bar{X}}_{h}\beta_{2\left(x_{h}\right)}+\rho_{h}}{{\hat{\bar{X}}}_{h}\beta_{2\left(x_{h}\right)}+\rho_{h}}\right]$
***β*_2_**(*p*_*h*_)	*C*_*ph*_	*β*_2_(*x*_*h*_)	*β*_1_(*x*_*h*_)	$T_{s_{4}}={\bar{y}}_{h}\sum_{h=1}^{k}W_{h}\left[\frac{{\bar{X}}_{h}\beta_{2\left(x_{h}\right)}+\beta_{1\left(x_{h}\right)}}{{\bar{\bar{X}}}_{h}\beta_{2\left(x_{h}\right)}+\beta_{1\left(x_{h}\right)}}\right]$
***β*_2_**(*p*_*h*_)	*C*_*ph*_	$C_{x_{h}}$	*β*_2_(*x*_*h*_)	$T_{s_{5}}={\bar{y}}_{h}\sum_{h=1}^{k}W_{h}\left[\frac{\bar{X}C_{x_{h}}+\beta_{2\left(x_{h}\right)}}{\left.{\hat{\bar{X}}}_{h}C_{x_{h}}+\beta_{2\left(x_{h}\right.}\right)}\right]$
***β*_2_**(*p*_*h*_)	*C*_*ph*_	*β*_1_(*p*_*h*_)	*β*_2_(*x*_*h*_)	$T_{s_{6}}={\bar{y}}_{h}\sum_{h=1}^{k}W_{h}\left[\frac{\bar{X}\beta_{1\left(p_{h}\right)}+\beta_{2\left(x_{h}\right)}}{{\hat{\bar{X}}}_{h}\beta_{1\left(p_{h}\right)}+\beta_{2\left(x_{h}\right)}}\right]$
***β*_2_**(*p*_*h*_)	*C*_*ph*_	*σ*_*h*_	*β*_2_(*x*_*h*_)	$T_{s_{7}}={\bar{y}}_{h}\sum_{h=1}^{k}W_{h}\left[\frac{\bar{X}\sigma_{h}+\beta_{2\left(x_{h}\right)}}{{\bar{\hat{X}}}_{h}\sigma_{h}+\beta_{2\left(x_{h}\right)}}\right]$
***β*_2_**(*p*_*h*_)	*C*_*ph*_	*β*_1_(*x*_*h*_)	*β*_2_(*x*_*h*_)	$T_{S_{8}}={\bar{y}}_{h}\sum_{h=1}^{k}W_{h}\left[\frac{{\bar{X}}_{1\left(x_{h}\right)}+\beta_{2\left(x_{h}\right)}}{{\hat{\bar{X}}}_{h}\beta_{1\left(x_{h}\right)}+\beta_{2\left(x_{h}\right)}}\right]$

**Table 2:** Particular situations for the proposed mixture estimator for *a* = 1.

*K*_1_	*K*_2_	*K*_3_	*K*_4_	Estimators
$C_{x_{h}}$	*β*_2_(*x*_*h*_)	*β*_2_(*p*_*h*_)	*C*_*ph*_	$T_{s_{9}}={\bar{y}}_{h}\sum_{h=1}^{k}W_{h}\left[\frac{P_{h}C_{x_{h}}+\beta_{2\left(x_{h}\right)}}{\hat{P}C_{x_{h}}+\beta_{2\left(x_{h}\right)}}\right]$
***β*_1_**(*p*_*h*_)	*β*_2_(*x*_*h*_)	*β*_2_(*p*_*h*_)	*C*_*ph*_	$T_{S_{10}}={\bar{y}}_{h}\sum_{h=1}^{k}W_{h}\left[\frac{P_{h}\beta_{1\left(p_{h}\right)}+\beta_{2\left(x_{h}\right)}}{\hat{P}\beta_{1\left(p_{h}\right)}+\beta_{2\left(x_{h}\right)}}\right]$
***σ_h_***	*β*_2_(*x*_*h*_)	*β*_2_(*p*_*h*_)	*C*_*ph*_	$T_{s_{11}}={\bar{y}}_{h}\sum_{h=1}^{k}W_{h}\left[\frac{P_{h}\sigma_{h}+\beta_{2\left(x_{h}\right)}}{\hat{P}\sigma_{h}+\beta_{2\left(x_{n}\right)}}\right]$
***ρ_h_***	*β*_2_(*x*_*h*_)	*β*_2_(*p*_*h*_)	*C*_*ph*_	$T_{S_{12}}={\bar{y}}_{h}\sum_{h=1}^{k}W_{h}\left[\frac{P\rho_{h}\rho_{h}+\beta_{2\left(x_{h}\right)}}{\hat{P}\rho_{h}+\beta_{2\left(x_{h}\right)}}\right]$
***β*_1_**(*x*_*h*_)	*β*_2_(*x*_*h*_)	*β*_2_(*p*_*h*_)	*C*_*ph*_	$T_{s_{13}}={\bar{y}}_{h}\sum_{h=1}^{k}W_{h}\left[\frac{P_{h}\beta_{1\left(x_{h}\right)}+\beta_{2\left(x_{n}\right)}}{\hat{P}\beta_{1\left(x_{h}\right)}+\beta_{2\left(x_{h}\right)}}\right]$
***β*_2_**(*x*_*h*_)	*C*_*x*_	*β*_2_(*p*_*h*_)	*C*_*ph*_	$T_{s_{14}}={\bar{y}}_{h}\sum_{h=1}^{k}W_{h}\left[\frac{P\beta_{2\left(x_{h}\right)}+C_{x_{h}}}{\hat{P}\beta_{2\left(x_{n}\right)}+C_{x_{h}}}\right]$
***β*_2_**(*x*_*h*_)	*β*_1_(*p*)	*β*_2_(*p*_*h*_)	*C*_*ph*_	$T_{S_{15}}={\bar{y}}_{h}\sum_{h=1}^{k}W_{h}\left[\frac{P\beta_{2\left(x_{h}\right)}+\beta_{1\left(p_{h}\right)}}{\hat{P}\beta_{2\left(x_{n}\right)}+\beta_{1\left(p_{h}\right)}}\right]$
***β*_2_**(*x*_*h*_)	σ	*β*_2_(*p*_*h*_)	*C*_*ph*_	$T_{S_{16}}={\bar{y}}_{h}\sum_{h=1}^{k}W_{h}\left[\frac{P\beta_{2\left(x_{h}\right)}+\sigma_{h}}{\hat{P}\beta_{2\left(x_{h}\right)}+\sigma_{h}}\right]$
***β*_2_**(*x*_*h*_)	*β*_1_(*x*)	*β*_2_(*p*_*h*_)	*C*_*ph*_	$T_{S_{17}}={\bar{y}}_{h}\sum_{h=1}^{k}W_{h}\left[\frac{P\beta_{2\left(x_{h}\right)}+\beta_{1\left(x_{h}\right)}}{\hat{P}\beta_{2\left(x_{h}\right)}+\beta_{1\left(x_{h}\right)}}\right]$

### Theoretical comparison

In this area, efficiency conditions are established by comparing the MSE of the suggested estimators with numerous existing estimators:

Referring to Eqs (2) and (29), $MSE\left({\hat{\mu }}_{yKS_{1},StRS}\right)>MSE\left(T_{KMst}\right)$ if and only if

$$\begin{array}{c} \sum_{h=1}^{k}\omega_{h}^{2}\lambda_{h}[S_{yh}^{2}-2RS_{syxh}+R^{2}S_{xh}^{2}]\\ >\sum_{h=1}^{k}\frac{N_{h}^{2}}{N^{2}}\left(\left(\lambda_{h}\left(S_{y_{h}}^{2}+\frac{{\bar{Y}}_{h}^{2}S_{x_{h}}^{2}}{{\bar{X}}_{U_{h}}^{2}}-\frac{2{\bar{Y}}_{h}S_{yx_{h}}}{{\bar{X}}_{U_{h}}}\right)+\lambda_{h}\frac{\left[-S_{yx_{h}}P_{U_{h}}+S_{yp_{h}}{\bar{X}}_{U_{h}}\right]+\frac{{\bar{Y}}_{h}}{{\bar{X}}_{U_{h}}}{\left[-S_{xp_{h}}{\bar{X}}_{U_{h}}+S_{x_{h}}^{2}P_{U_{h}}\right]}^{2}}{S_{p_{h}}^{2}{\bar{X}}_{U_{h}}^{2}+S_{x_{h}}^{2}P_{U_{h}}^{2}+2S_{xp_{h}}{\bar{X}}_{U_{h}}.P_{U_{h}}}\right)\right) \end{array}.$$ (31)

Referring to Eqs (4) and (29), $MSE\left({\hat{\mu }}_{yKS_{2},StRS}\right)>MSE\left(T_{KMst}\right)$ if and only if

$$\begin{array}{c} \sum_{h=1}^{k}\omega_{h}^{2}\lambda_{h}[S_{yh}^{2}-2RS_{syxh}+R^{2}S_{xh}^{2}]\\ >\sum_{h=1}^{k}\frac{N_{h}^{2}}{N^{2}}\left(\left(\lambda_{h}\left(S_{y_{h}}^{2}+\frac{{\bar{Y}}_{h}^{2}S_{x_{h}}^{2}}{{\bar{X}}_{U_{h}}^{2}}-\frac{2{\bar{Y}}_{h}S_{yx_{h}}}{{\bar{X}}_{U_{h}}}\right)+\lambda_{h}\frac{\left[-S_{yx_{h}}P_{U_{h}}+S_{yp_{h}}{\bar{X}}_{U_{h}}\right]+\frac{{\bar{Y}}_{h}}{{\bar{X}}_{U_{h}}}{\left[-S_{xp_{h}}{\bar{X}}_{U_{h}}+S_{x_{h}}^{2}P_{U_{h}}\right]}^{2}}{S_{p_{h}}^{2}{\bar{X}}_{U_{h}}^{2}+S_{x_{h}}^{2}P_{U_{h}}^{2}+2S_{xp_{h}}{\bar{X}}_{U_{h}}.P_{U_{h}}}\right)\right) \end{array}.$$ (32)

Referring to Eqs (6) and (29), $MSE\left({\hat{\mu }}_{yKS_{3},StRS}\right)>MSE\left(T_{KMst}\right)$ if and only if

$$\begin{array}{c} \sum_{h=1}^{k}\omega_{h}^{2}\lambda_{h}[S_{yh}^{2}-2RS_{syxh}+R^{2}S_{xh}^{2}]\\ >\sum_{h=1}^{k}\frac{N_{h}^{2}}{N^{2}}\left(\left(\lambda_{h}\left(S_{y_{h}}^{2}+\frac{{\bar{Y}}_{h}^{2}S_{x_{h}}^{2}}{{\bar{X}}_{U_{h}}^{2}}-\frac{2{\bar{Y}}_{h}S_{yx_{h}}}{{\bar{X}}_{U_{h}}}\right)+\lambda_{h}\frac{\left[-S_{yx_{h}}P_{U_{h}}+S_{yp_{h}}{\bar{X}}_{U_{h}}\right]+\frac{{\bar{Y}}_{h}}{{\bar{X}}_{U_{h}}}{\left[-S_{xp_{h}}{\bar{X}}_{U_{h}}+S_{x_{h}}^{2}P_{U_{h}}\right]}^{2}}{S_{p_{h}}^{2}{\bar{X}}_{U_{h}}^{2}+S_{x_{h}}^{2}P_{U_{h}}^{2}+2S_{xp_{h}}{\bar{X}}_{U_{h}}.P_{U_{h}}}\right)\right) \end{array}.$$ (33)

Referring to Eqs (8) and (29), $MSE\left({\hat{\mu }}_{yKS_{4},StRS}\right)>MSE\left(T_{KMst}\right)$ if and only if

$$\begin{array}{c} \sum_{h=1}^{k}\omega_{h}^{2}\lambda_{h}[S_{yh}^{2}-2RS_{syxh}+R^{2}S_{xh}^{2}]\\ >\sum_{h=1}^{k}\frac{N_{h}^{2}}{N^{2}}\left(\left(\lambda_{h}\left(S_{y_{h}}^{2}+\frac{{\bar{Y}}_{h}^{2}S_{x_{h}}^{2}}{{\bar{X}}_{U_{h}}^{2}}-\frac{2{\bar{Y}}_{h}S_{yx_{h}}}{{\bar{X}}_{U_{h}}}\right)+\lambda_{h}\frac{\left[-S_{yx_{h}}P_{U_{h}}+S_{yp_{h}}{\bar{X}}_{U_{h}}\right]+\frac{{\bar{Y}}_{h}}{{\bar{X}}_{U_{h}}}{\left[-S_{xp_{h}}{\bar{X}}_{U_{h}}+S_{x_{h}}^{2}P_{U_{h}}\right]}^{2}}{S_{p_{h}}^{2}{\bar{X}}_{U_{h}}^{2}+S_{x_{h}}^{2}P_{U_{h}}^{2}+2S_{xp_{h}}{\bar{X}}_{U_{h}}.P_{U_{h}}}\right)\right) \end{array}.$$ (34)

Referring to Eqs (10) and (29), $MSE\left({\hat{\mu }}_{yZ_{1},StRS}\right)>MSE\left(T_{KMst}\right)$ if and only if

$$\begin{array}{c} \sum_{h=1}^{k}\omega_{h}^{2}\lambda_{h}[S_{yh}^{2}-2RS_{syph}+R^{2}S_{ph}^{2}]\\ >\sum_{h=1}^{k}\frac{N_{h}^{2}}{N^{2}}\left(\left(\lambda_{h}\left(S_{y_{h}}^{2}+\frac{{\bar{Y}}_{h}^{2}S_{x_{h}}^{2}}{{\bar{X}}_{U_{h}}^{2}}-\frac{2{\bar{Y}}_{h}S_{yx_{h}}}{{\bar{X}}_{U_{h}}}\right)+\lambda_{h}\frac{\left[-S_{yx_{h}}P_{U_{h}}+S_{yp_{h}}{\bar{X}}_{U_{h}}\right]+\frac{{\bar{Y}}_{h}}{{\bar{X}}_{U_{h}}}{\left[-S_{xp_{h}}{\bar{X}}_{U_{h}}+S_{x_{h}}^{2}P_{U_{h}}\right]}^{2}}{S_{p_{h}}^{2}{\bar{X}}_{U_{h}}^{2}+S_{x_{h}}^{2}P_{U_{h}}^{2}+2S_{xp_{h}}{\bar{X}}_{U_{h}}.P_{U_{h}}}\right)\right) \end{array}.$$ (35)

Referring to Eqs (12) and (29), $MSE\left({\hat{\mu }}_{yZ_{2},StRS}\right)>MSE\left(T_{KMst}\right)$ if and only if

$$\begin{array}{c} \sum_{h=1}^{k}\omega_{h}^{2}\lambda_{h}[S_{yh}^{2}-2RS_{syph}+R^{2}S_{ph}^{2}]\\ >\sum_{h=1}^{k}\frac{N_{h}^{2}}{N^{2}}\left(\left(\lambda_{h}\left(S_{y_{h}}^{2}+\frac{{\bar{Y}}_{h}^{2}S_{x_{h}}^{2}}{{\bar{X}}_{U_{h}}^{2}}-\frac{2{\bar{Y}}_{h}S_{yx_{h}}}{{\bar{X}}_{U_{h}}}\right)+\lambda_{h}\frac{\left[-S_{yx_{h}}P_{U_{h}}+S_{yp_{h}}{\bar{X}}_{U_{h}}\right]+\frac{{\bar{Y}}_{h}}{{\bar{X}}_{U_{h}}}{\left[-S_{xp_{h}}{\bar{X}}_{U_{h}}+S_{x_{h}}^{2}P_{U_{h}}\right]}^{2}}{S_{p_{h}}^{2}{\bar{X}}_{U_{h}}^{2}+S_{x_{h}}^{2}P_{U_{h}}^{2}+2S_{xp_{h}}{\bar{X}}_{U_{h}}.P_{U_{h}}}\right)\right) \end{array}.$$ (36)

Referring to Eqs (14) and (29), $MSE\left({\hat{\mu }}_{yZ_{3},StRS}\right)>MSE\left(T_{KMst}\right)$ if and only if

$$\begin{array}{c} \sum_{h=1}^{k}\omega_{h}^{2}\lambda_{h}[S_{yh}^{2}-2RS_{syph}+R^{2}S_{ph}^{2}]\\ >\sum_{h=1}^{k}\frac{N_{h}^{2}}{N^{2}}\left(\left(\lambda_{h}\left(S_{y_{h}}^{2}+\frac{{\bar{Y}}_{h}^{2}S_{x_{h}}^{2}}{{\bar{X}}_{U_{h}}^{2}}-\frac{2{\bar{Y}}_{h}S_{yx_{h}}}{{\bar{X}}_{U_{h}}}\right)+\lambda_{h}\frac{\left[-S_{yx_{h}}P_{U_{h}}+S_{yp_{h}}{\bar{X}}_{U_{h}}\right]+\frac{{\bar{Y}}_{h}}{{\bar{X}}_{U_{h}}}{\left[-S_{xp_{h}}{\bar{X}}_{U_{h}}+S_{x_{h}}^{2}P_{U_{h}}\right]}^{2}}{S_{p_{h}}^{2}{\bar{X}}_{U_{h}}^{2}+S_{x_{h}}^{2}P_{U_{h}}^{2}+2S_{xp_{h}}{\bar{X}}_{U_{h}}.P_{U_{h}}}\right)\right) \end{array}.$$ (37)

Referring to Eqs (16) and (29), $MSE\left({\hat{\mu }}_{yZ_{4},StRS}\right)>MSE\left(T_{KMst}\right)$ if and only if

$$\begin{array}{c} \sum_{h=1}^{k}\omega_{h}^{2}\lambda_{h}[S_{yh}^{2}-2RS_{syph}+R^{2}S_{ph}^{2}]\\ >\sum_{h=1}^{k}\frac{N_{h}^{2}}{N^{2}}\left(\left(\lambda_{h}\left(S_{y_{h}}^{2}+\frac{{\bar{Y}}_{h}^{2}S_{x_{h}}^{2}}{{\bar{X}}_{U_{h}}^{2}}-\frac{2{\bar{Y}}_{h}S_{yx_{h}}}{{\bar{X}}_{U_{h}}}\right)+\lambda_{h}\frac{\left[-S_{yx_{h}}P_{U_{h}}+S_{yp_{h}}{\bar{X}}_{U_{h}}\right]+\frac{{\bar{Y}}_{h}}{{\bar{X}}_{U_{h}}}{\left[-S_{xp_{h}}{\bar{X}}_{U_{h}}+S_{x_{h}}^{2}P_{U_{h}}\right]}^{2}}{S_{p_{h}}^{2}{\bar{X}}_{U_{h}}^{2}+S_{x_{h}}^{2}P_{U_{h}}^{2}+2S_{xp_{h}}{\bar{X}}_{U_{h}}.P_{U_{h}}}\right)\right) \end{array}.$$ (38)

Referring to Eqs (18) and (29), $MSE\left({\hat{\mu }}_{yZ_{5},StRS}\right)>MSE\left(T_{KMst}\right)$ if and only if

$$\begin{array}{c} \sum_{h=1}^{k}\omega_{h}^{2}\lambda_{h}[S_{yh}^{2}-2RS_{syph}+R^{2}S_{ph}^{2}]\\ >\sum_{h=1}^{k}\frac{N_{h}^{2}}{N^{2}}\left(\left(\lambda_{h}\left(S_{y_{h}}^{2}+\frac{{\bar{Y}}_{h}^{2}S_{x_{h}}^{2}}{{\bar{X}}_{U_{h}}^{2}}-\frac{2{\bar{Y}}_{h}S_{yx_{h}}}{{\bar{X}}_{U_{h}}}\right)+\lambda_{h}\frac{\left[-S_{yx_{h}}P_{U_{h}}+S_{yp_{h}}{\bar{X}}_{U_{h}}\right]+\frac{{\bar{Y}}_{h}}{{\bar{X}}_{U_{h}}}{\left[-S_{xp_{h}}{\bar{X}}_{U_{h}}+S_{x_{h}}^{2}P_{U_{h}}\right]}^{2}}{S_{p_{h}}^{2}{\bar{X}}_{U_{h}}^{2}+S_{x_{h}}^{2}P_{U_{h}}^{2}+2S_{xp_{h}}{\bar{X}}_{U_{h}}.P_{U_{h}}}\right)\right) \end{array}.$$ (39)

### A simulation study

We conducted a comprehensive simulation study to assess the proposed estimator’s effectiveness. This involved comparing the performance of our estimator with that of several alternative estimators under StRS conditions. The percent relative efficiency (PRE) served as the criterion for evaluating estimator performance. We followed the steps outlined below to compute the PREs for our proposed estimator under StRS.

1. A simulated population comprising 1500 observations is established for the study variable Y. X serves as an auxiliary variable, while P represents an auxiliary attribute. Random selections are made from Normal and Uniform (symmetric), Gamma, and Weibull (asymmetric) distributions across 10,000 samples of specified sizes. Table 3 outlines the methodology for utilizing the Bernoulli distribution with specific parameter values to generate the auxiliary attribute. Moreover, population size is considered as *N* = *N*_1_ + *N*_2_ = 800 + 700 = 1500.
2. The given proportional allocation formula is used to determine the sample size from the stratum.

$$n_{h}/n=N_{h}/N\;or\;n_{h}⇌\frac{n}{N}N_{h}=nW_{h}$$

Using StRS, the population was divided into two strata: 800 and 700. Further, 10,000 random samples of size 20 are drawn by taking 12 units from stratum one and 8 units from stratum two. Next, 10,000 random samples of size 50 are drawn by taking 30 units from stratum one and 20 units from stratum two. Similarly, using the proportional allocation scheme, the next 10,000 random samples of size 80 are obtained by selecting 50 units from stratum one and 30 units from stratum two. Next, 10,000 random samples of size 200 are drawn by taking 120 units from stratum one and 80 units from stratum two. Further next, 10,000 random samples of size 400 are drawn by taking 280 units from stratum one and 120 units from stratum two. The results are shown in Table 4.

1. The PREs of estimators are calculated by using the following expression:

$$PRE=\frac{MSE\left({\hat{\mu }}_{y,StRS}\right)}{MSE\left({\hat{\mu }}_{y,i}\right)}\times 100$$ (40)

where ${\hat{a}}_{y,StRS}=\sum_{i=1}^{k}\frac{N_{h}}{N}\left[\frac{{\bar{y}}_{h}}{{\bar{x}}_{h}}\bar{X}\right]$ and “i” stands for the estimator, whose performance needs to be compared. To check the efficiency of proposed estimator the expression of MSE given in Eq (30) has been utilized.

**Table 3:** Simulated auxiliary variables and attributes.

	Distribution Parameters	Normal			Uniform			Gamma			Weibull		
		τ	*Y*	*X*	τ	*Y*	*X*	τ	*Y*	*X*	τ	*Y*	*X*
Stratum-I *N*_1h_ = 800	*n*	1			1			1			1		
	*P*	0.5			0.3			0.5			0.7		
	Shape					0	0.1		2	2		2	2
	Scale		600	1200		1	1.1		1	1		1	1
	Location		550	850									
Stratum-II *N*_2h_ = 700	*n*	1			1			1			1		
	*P*	0.5			0.7			0.5			0.3		
	Shape					0	0.1		2.5	2.5		4	4
	Scale		400	1000		1	1.1		1.5	1.5		1	1
	Location		500	900									

**Table 4:** Two homogeneous subgroups of the population.

Prop. Allocation	Stratum-I	Stratum-II
	*n*_1h_ = 12,30,50,120,280	*n*_2h_ = 8,20,30,80,120
*N*_*h*_	800	700
*W*_*h*_	800/1500	700/1500
*nW*_*h*_	20*800/1500, 50*800/1500, 80*800/1500, 120*800/1500, 280*800/1500	8*700/1500, 20*700/1500, 30*700/1500, 80*700/1500, 120*700/1500

**Fig 1:**
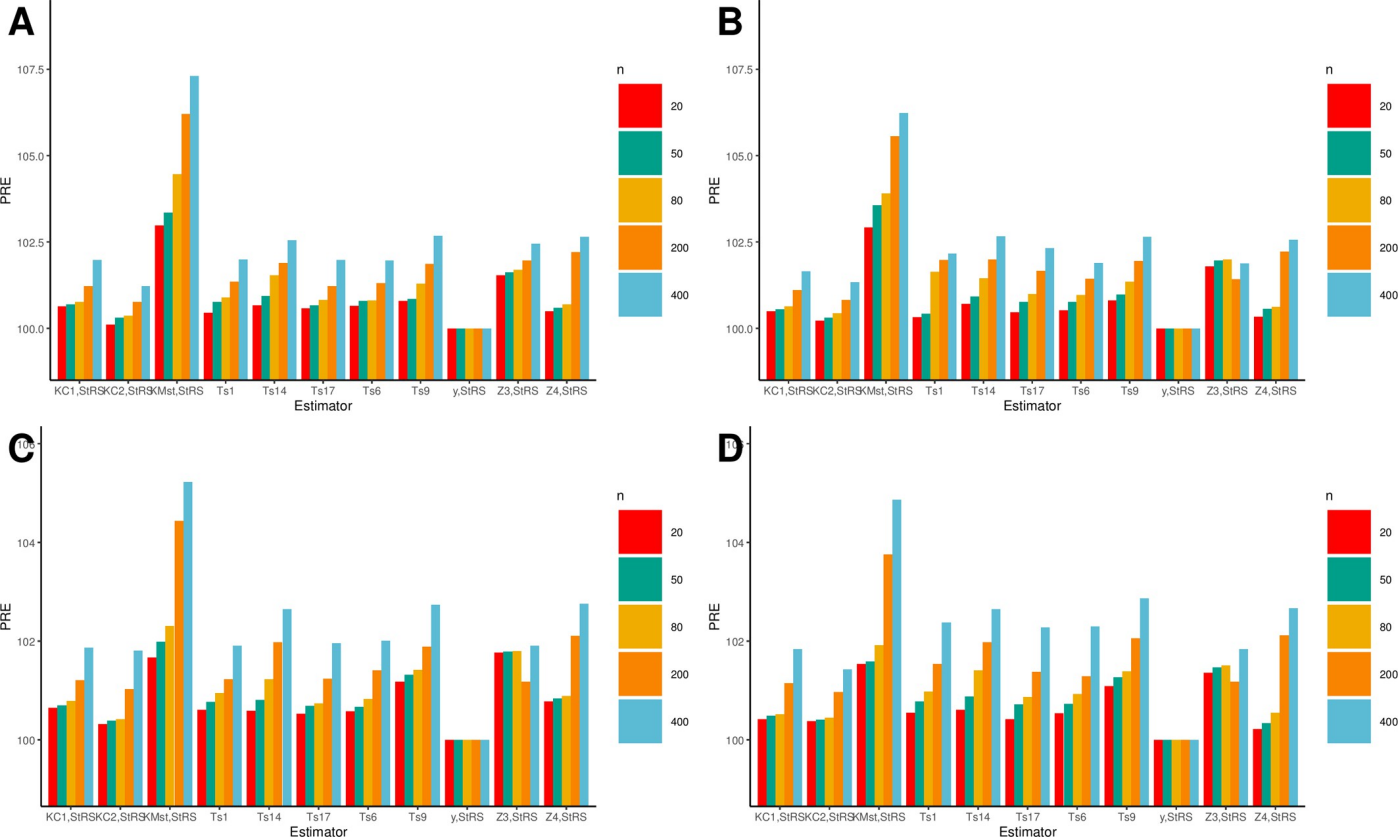
A comparison between the proposed estimator and competitive estimators under the following distributions. (A) normal, (B) uniform, (C) Gamma, and (D) Weibull.

Tables 5 and 6 present the PREs of the proposed estimator in comparison to those of competing estimators. We also assessed the impact of sample size on MSEs and employed sample sizes of 20, 50,80, 200, and 400. Under Normal distribution, for *n* = 20,50,80,200 and 400,the proposed generalized mixture estimator $T_{KM_{st}}$ the highest PRE’s are 102.98, 103.35, 104.46, 106.21, and 107.31 respectively. Similarly, under the Uniform distribution, for *n* = 20,50,80,200 and 400, the proposed generalized mixture estimator $T_{KM_{st}}$ the highest PREs were reported as 102.92, 103.56,103.91, 105.56, and 106.23 respectively. Moreover, similar results are evident in Table 6, particularly under the Gamma and Weibull distributions, the proposed generalized mixture estimator $T_{KM_{st}}$ demonstrates significantly superior PRE values when compared to the other estimators under consideration. Additionally, it was noted that the percent relative efficiency (PRE) values demonstrated an increase as sample sizes increased, as illustrated in Fig 1.

**Table 5:** Proposed generalized mixture estimators’ percent relative efficiencies (PREs) and comparative estimators’ PREs for Normal and Uniform distributions.

Normal						Uniform				
**Estimators**	*n* = 20	*n* = 50	*n* = 80	*n* = 200	*n* = 400	*n* = 20	*n* = 50	*n* = 80	*n* = 200	*n* = 400
${\hat{\mu }}_{y,StRS}$	100	100	100	100	100	100	100	100	100	100
${\hat{\mu }}_{yKC_{\text{1}},StRS}$	100.64	100.69	100.77	101.23	101.98	100.50	100.55	100.64	101.11	101.65
${\hat{\mu }}_{yKC_{\text{2}},StRS}$	100.11	100.31	100.37	100.76	101.23	100.23	100.31	100.44	100.82	101.34
${\hat{\mu }}_{yKC_{\text{3}},StRS}$	100.40	100.49	100.52	100.89	101.34	100.17	100.27	100.39	100.77	101.11
${\hat{\mu }}_{yKC_{\text{4}},StRS}$	101.37	101.48	101.55	101.99	102.01	100.62	100.75	100.77	101.31	101.99
${\hat{\mu }}_{yZ_{\text{1}},StRS}$	100.13	100.20	100.36	101.21	101.56	100.69	100.76	100.80	101.24	102.01
${\hat{\mu }}_{yZ_{\text{2}},StRS}$	100.56	100.65	100.68	100.87	101.56	100.93	101.13	101.66	102.14	102.76
${\hat{\mu }}_{yZ_{3},StRS}$	101.45	101.53	101.60	101.97	102.45	100.80	100.91	100.98	101.43	101.88
${\hat{\mu }}_{yZ_{4},StRS}$	101.54	101.63	101.69	102.21	102.65	101.79	101.96	102.00	102.22	102.56
${\hat{\mu }}_{yZ_{5},StRS}$	100.49	100.59	100.70	101.02	101.45	100.34	100.56	100.62	101.04	101.76
$T_{KM_{st}}$	**102.98**	**103.35**	**104.46**	**106.21**	**107.31**	**102.92**	**103.56**	**103.91**	**105.56**	**106.23**
$T_{s_{1}}$	100.45	100.76	100.89	101.35	101.99	100.32	100.43	101.64	101.98	102.16
$T_{s_{2}}$	100.65	100.68	100.76	101.02	102.21	100.44	100.76	100.90	101.21	101.98
$T_{s_{3}}$	100.49	100.70	100.76	101.12	101.96	100.55	100.73	100.79	101.13	101.87
$T_{s_{4}}$	100.35	100.44	100.65	101.02	101.56	100.48	100.51	100.73	101.22	101.86
$T_{s_{5}}$	100.44	100.63	100.75	101.22	101.86	100.34	100.62	100.82	101.09	101.78
$T_{s_{6}}$	100.65	100.79	100.81	101.31	101.97	100.52	100.77	100.96	101.44	101.89
$T_{s_{7}}$	100.33	100.49	100.57	100.97	101.44	100.21	100.53	100.63	101.17	101.95
$T_{s_{8}}$	100.39	100.56	100.68	101.04	101.68	100.46	100.69	100.95	101.54	102.01
$T_{s_{9}}$	100.79	100.85	101.29	101.87	102.68	100.81	100.98	101.35	101.95	102.65
$T_{s_{10}}$	100.65	100.75	100.77	101.11	101.77	100.33	100.65	100.81	101.24	101.84
$T_{s_{11}}$	100.53	100.69	100.74	101.24	101.95	100.41	100.57	100.83	101.31	101.92
$T_{s_{12}}$	100.61	100.77	100.95	101.35	102.34	100.57	100.70	100.99	101.56	102.21
$T_{s_{13}}$	100.34	100.79	100.88	101.54	102.01	100.38	100.66	100.78	101.33	101.83
$T_{s_{14}}$	100.67	100.94	101.54	101.89	102.55	100.71	100.93	101.45	101.99	102.67
$T_{s_{15}}$	100.44	100.85	100.86	101.21	101.88	100.48	100.90	101.00	101.76	102.65
$T_{s_{16}}$	100.42	100.68	100.81	101.08	101.79	100.53	100.72	100.90	101.27	102.43
$T_{s_{17}}$	100.58	100.67	100.83	101.23	101.98	100.46	100.77	100.99	101.67	102.32

**Table 6:** Proposed generalized mixture estimators’ percent relative efficiencies (PREs) and comparative estimators’ PREs for Gamma and Weibull distributions.

Normal						Uniform				
**Estimators**	*n* = 20	*n* = 50	*n* = 80	*n* = 200	*n* = 400	*n* = 20	*n* = 50	*n* = 80	*n* = 200	*n* = 400
${\hat{\mu }}_{y,StRS}$	100	100	100	100	100	100	100	100	100	100
${\hat{\mu }}_{yKC_{\text{1}},StRS}$	100.65	100.70	100.79	101.21	101.87	100.42	100.49	100.52	101.15	101.84
${\hat{\mu }}_{yKC_{2},StRS}$	100.32	100.39	100.42	101.03	101.81	100.38	100.41	100.45	100.97	101.43
${\hat{\mu }}_{yKC_{3},StRS}$	100.58	100.70	100.71	101.11	101.64	100.92	101.00	101.20	101.98	102.31
${\hat{\mu }}_{yKC_{4},StRS}$	100.63	100.75	100.81	101.07	101.77	100.95	100.99	101.18	101.88	102.65
${\hat{\mu }}_{yZ_{\text{1}},StRS}$	100.71	100.79	100.87	101.32	101.83	100.88	100.90	100.93	101.65	102.03
${\hat{\mu }}_{yZ_{\text{2}},StRS}$	100.19	100.35	100.44	100.97	101.32	100.67	100.71	100.77	101.34	102.06
${\hat{\mu }}_{yZ_{\text{3}},StRS}$	100.67	100.80	100.88	101.18	101.91	100.65	100.68	100.72	101.18	101.84
${\hat{\mu }}_{yZ_{\text{4}},StRS}$	100.77	101.79	101.82	102.11	102.76	101.36	101.47	101.51	102.12	102.67
${\hat{\mu }}_{yZ_{\text{5}},StRS}$	100.78	100.84	100.89	101.15	101.46	100.22	100.34	100.55	101.06	101.76
$T_{KM_{st}}$	**101.67**	**101.99**	**102.31**	**104.44**	**105.23**	**101.54**	**101.59**	**101.92**	**103.76**	**104.87**
$T_{s_{\text{1}}}$	100.61	100.77	100.95	101.23	101.91	100.55	100.78	100.98	101.54	102.38
$T_{s_{2}}$	100.34	100.79	100.88	101.33	101.90	100.43	100.71	100.92	101.33	102.01
$T_{s_{3}}$	100.67	100.74	100.86	101.17	101.87	100.57	100.80	100.98	101.54	102.41
$T_{s_{4}}$	100.44	100.85	100.86	101.34	102.00	100.38	100.77	100.99	101.65	102.18
$T_{s_{5}}$	100.42	100.68	100.81	101.29	101.93	100.41	100.63	100.87	101.47	102.43
$T_{s_{6}}$	100.58	100.67	100.83	101.41	102.01	100.54	100.73	100.93	101.29	102.30
$T_{s_{7}}$	100.45	100.76	100.89	101.27	101.92	100.51	100.67	100.91	101.36	102.22
$T_{s_{8}}$	100.65	100.68	100.76	100.99	101.59	100.58	100.81	100.90	101.56	102.19
$T_{s_{9}}$	101.18	101.32	101.42	101.89	102.74	101.09	101.27	101.39	102.06	102.87
$T_{s_{10}}$	100.35	100.44	100.65	101.55	102.04	100.37	100.53	100.82	101.66	102.15
$T_{s_{11}}$	100.44	100.63	100.75	101.34	101.99	100.39	100.69	100.83	101.27	101.99
$T_{s_{12}}$	100.65	100.79	100.81	101.45	102.65	100.68	100.82	100.96	101.67	102.43
$T_{s_{13}}$	100.33	100.49	100.57	101.43	102.32	100.23	100.44	100.81	101.53	102.44
$T_{s_{14}}$	100.59	100.81	101.23	101.98	102.65	100.61	100.88	101.41	101.98	102.65
$T_{s_{15}}$	100.49	100.70	100.76	101.44	102.03	100.32	100.53	100.88	101.73	102.34
$T_{s_{16}}$	100.65	100.75	100.77	101.31	102.33	100.43	100.68	100.74	101.54	102.11
$T_{s_{17}}$	100.53	100.69	100.74	101.24	101.96	100.42	100.72	100.87	101.38	102.28

### Exploring the Best-Fitted distribution

This section explores the most appropriate probability distribution for the proposed generalized mixture estimator across various sample sizes using EasyFit [30]. EasyFit employs two methods, namely the Kolmogorov-Smirnov and Anderson-Darling tests, for this purpose. The auxiliary variables are generated from a normal distribution, while the auxiliary attribute is generated from a binomial distribution using R language (version 4.2.2), with parameters specified in Table 7. We select one thousand samples of sizes n = 20, n = 50, and n = 80 and then construct the sampling distribution of the proposed estimator.

**Table 7:** Setting up additional variables and characteristics for the simulation study.

Variables and Attributes	*N*	Stratum-I					Stratum-II				
		*N*_1h_ = 800					*N*_2h_ = 700				
		Parameters									
		*μ*	*σ*	*ρ*_*xy*_	*n*	*p*	*μ*	*σ*	*ρ*_*xy*_	*n*	*p*
***Y***	1500	550	600	0.99			500	400	0.88		
***X***	1500	850	1200	0.99			900	1000	0.88		
***P***	1500				2	0.5				2	0.5

**Fig 2:**
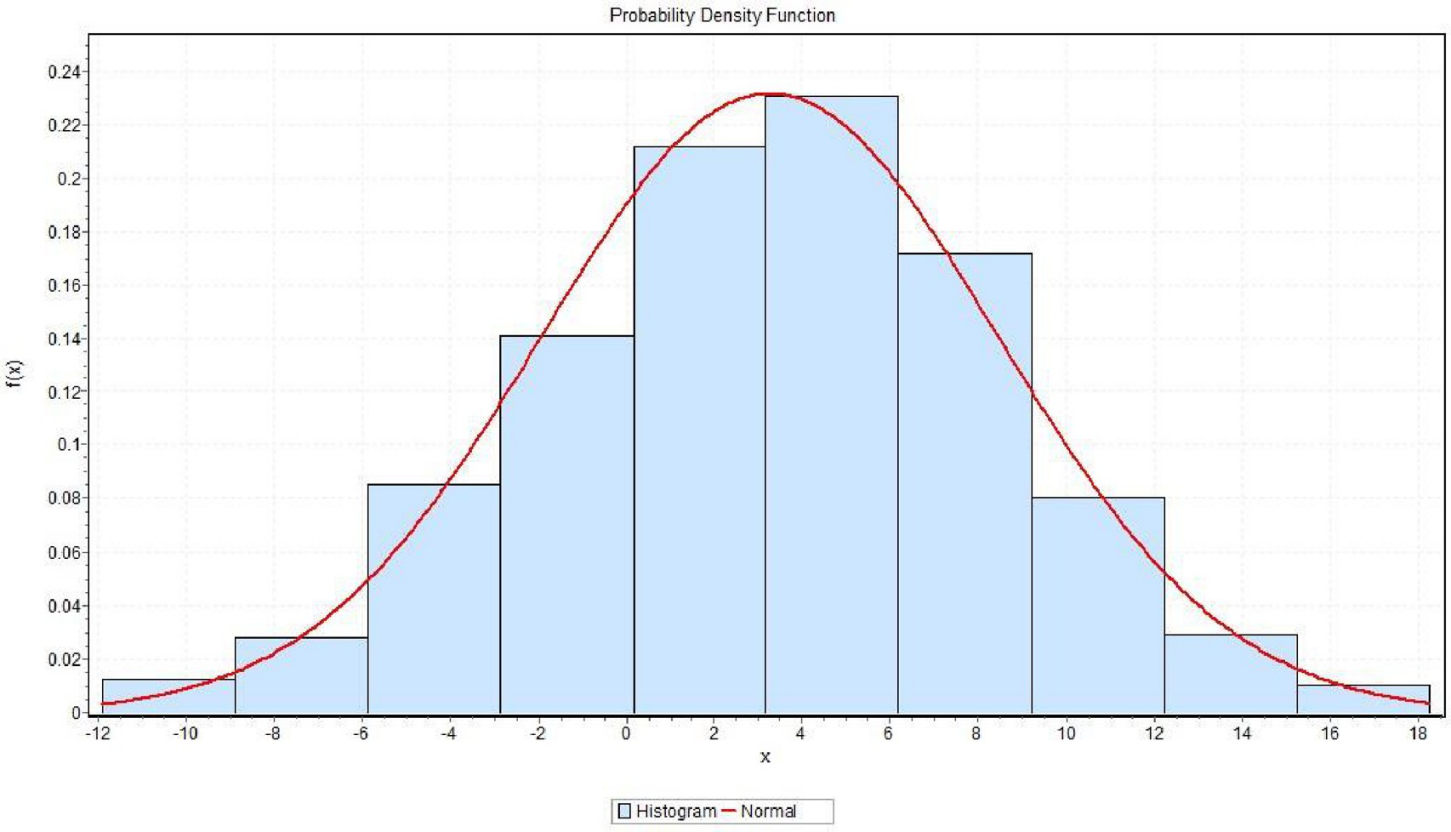
Probability distribution of generalized proposed mixture estimator.

As depicted in Fig 2, the proposed generalized mixture estimator conforms to the Normal distribution, with the scale parameter and location parameter computed as 3.13 and 5.30, respectively.

### Exploring the distribution of the proposed estimator for different sample sizes

This section examines how the suggested generalized mixture estimator’s distribution behaves for different sample sizes.

Table 8 displays the probability distribution of the estimator for *n* = 20, 50, and 80. When the initial data is derived from the Normal distribution for a sample size of *n* = 20, the proposed estimator follows the Cauchy distribution, with the cutoff point being *n* = 35, for which the proposed estimator’s probability distribution is converged to be Normal. Hence, the proposed estimator follows the normal distribution for n = 50 and 80.

**Table 8:** Probability distribution of estimator for different sample sizes.

Distribution of the Variable	Sample Sizes	Distribution of Estimators	
**Normal**	20	Cauchy (*n* < 35)	Normal (*n* ≥ 35)
	50	Normal	
	80	Normal	

Table 9 illustrates the probability distributions of the proposed estimator against each value of n. The Kolmogorov–Smirnov results indicate that the p-values exceed 5%, supporting the hypotheses (H_0_) that the data adhere to the specified distribution.

**Table 9:** Probability distribution of estimator for each sample size.

*n*	*p-value (KS test)*	*Distribution of Estimator*	*n*	*p-value (KS test)*	*Distribution of Estimator*	*n*	*p-value (KS test)*	*Distribution of Estimator*
20	0.691	Cauchy	40	0.496	Normal	61	0.569	Normal
21	0.662	Cauchy	42	0.661	Normal	62	0.211	Normal
22	0.667	Cauchy	43	0.372	Normal	63	0.162	Normal
23	0.692	Cauchy	44	0.451	Normal	64	0.544	Normal
24	0.656	Cauchy	45	0.714	Normal	65	0.692	Normal
25	0.545	Cauchy	46	0.685	Normal	66	0.734	Normal
26	0.589	Cauchy	47	0.699	Normal	67	0.181	Normal
27	0.576	Cauchy	48	0.127	Normal	68	0.812	Normal
28	0.566	Cauchy	49	0.754	Normal	69	0.732	Normal
29	0.432	Cauchy	50	0.671	Normal	70	0.541	Normal
30	0.591	Cauchy	51	0.651	Normal	71	0.417	Normal
31	0.612	Cauchy	52	0.161	Normal	72	0.568	Normal
32	0.655	Cauchy	53	0.362	Normal	73	0.485	Normal
33	0.212	Cauchy	54	0.324	Normal	74	0.721	Normal
34	0.426	Cauchy	55	0.712	Normal	75	0.547	Normal
35	0.525	Normal	56	0.621	Normal	76	0.167	Normal
36	0.769	Normal	57	0.676	Normal	77	0.651	Normal
37	0.616	Normal	58	0.269	Normal	78	0.611	Normal
38	0.321	Normal	59	0.665	Normal	79	0.232	Normal
39	0.263	Normal	60	0.455	Normal	80	0.423	Normal

### Illustrative examples

In this section, we present two real-life examples to illustrate the practical application of the proposed estimator. The distribution of study and auxiliary variables in each of the two data sets is discovered using EasyFit version 5.5 professional. The details of each of the data sets are given below.

### Data-I: Tumor data

Data-I has been taken from Andersen, Borgan [31]. We are interested in estimating the average thickness of the tumor by including auxiliary information. The data consists of 205 entities. StRS has been used, and the given variables and attributes have been considered. ***Y*:** Thickness of tumor (mm), ***X***: Age of patient (at operation time), and ***P***: Gender (0 = Male, 1 = Female) are used as auxiliary variable and attribute. The variable ’whether a patient was ulcerated or not’ has been used for stratification purposes. Those patients who are not ulcerated are placed in stratum 1, while stratum 2 consists of the remaining patients. In Table 10, the parameters of the data have been presented.

**Table 10:** Descriptive details of study variable *Y*, auxiliary variable *X*, and attribute *P*.

Variables	Strata	Size	Mean	Variance	Minimum	Maximum
***Y***	1	115	1.8113	4.778	0.1	14.66
	2	90	4.3363	10.3382	0.16	17.42
***X***		205	52.463	277.95	4	95
***P***		205	0.385	0.238	0	1

The population of size 205 is split into two strata, with sizes 115 and 90, respectively, as Table 10 illustrates. The proportional allocation scheme has been utilized to select random samples of sizes 12, 30, 50, and 8, 20, 30, and PREs are computed. In stratum 1, the average thickness of the tumor is 1.8113, while in stratum 2, its value is 4.3433. The variations in the tumor thickness in strata 1 and 2 are 4.7364 and 10.4244, respectively. The average age is 52.463, and the average attribute gender is 0.385. The variation in age is 277.95, and in gender, it is 0.238.

### Data-II: Wages data

In this example, the data set obtained from Bierens and Ginther [32] is about wages of employees in the United States of America (USA) is considered, where wage (in dollars per week) is the study variable ***Y*,** number of years of education is the auxiliary variable ***X***, and living in the standard metropolitan statistical area (SMSA) (0 = Not lives, 1 = if lives) is used as the auxiliary attribute ***P***. In Table 11, the parameters of the data have been presented.

**Table 11:** Descriptive details of study variable *Y*, auxiliary variable *X*, and attribute *P*.

Variables	Strata	Size	Mean	Variance	Minimum	Maximum
***Y***	1	25631	640.2	197379.9	50.4	18777.2
	2	2524	233.73	139839.9	50.5	9259.26
***X***		28155	13.067	8.408	0	18
***P***		28155	0.7435	0.1907	0	1

Table 11 shows that the original data set contained 28155 observations. The observations are divided into two strata of sizes 25631 and 2524, respectively. The proportional allocation scheme has been utilized to select random samples of sizes 12, 30, 50, and 8, 20, 30, and PREs is computed. In stratum 1, the average wage is 640.2, while in stratum 2, the average wage is 233.73. In strata 1, the variation in wage is 197379.9, and in strata 2, its value is 139839.9. The average number of years of education is 13.067, and the average SMSA is 0.7435. Finally, the variation in the number of years of education is 8.408, and in SMSA, the variation is 0.1907.

**Fig 3:**
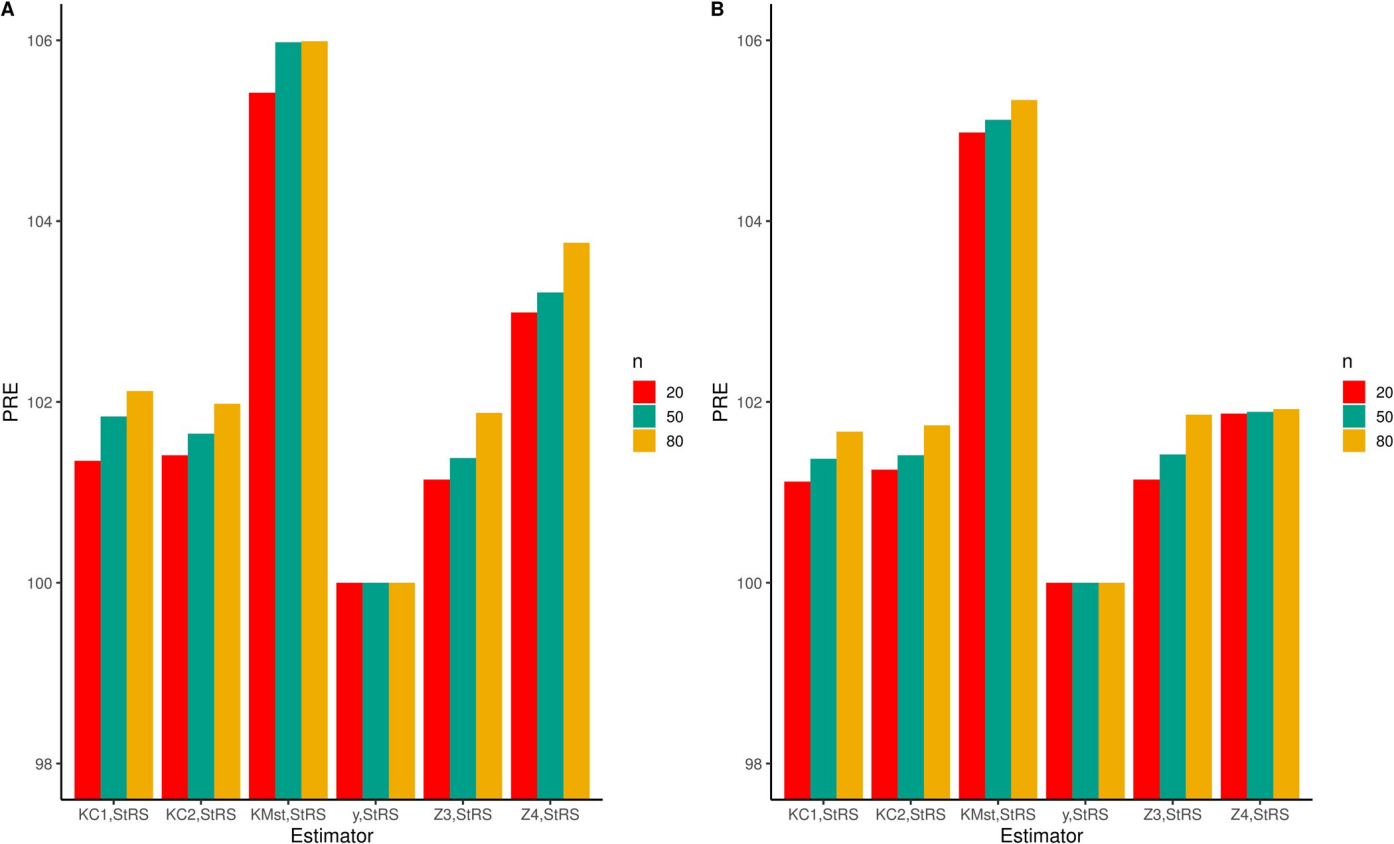
A comparison between the proposed estimator and competitive estimators for real-life datasets. (A) Data-I and (B) Data-II.

Tables 12 and 13 shows the necessary calculations (estimated means and PRE’s) for proposed and comparative estimators with respect to StRS for those two real-life datasets mentioned. We used 20, 50, and 80 sample sizes for each data set. In dataset I, $T_{KM_{st}}$ with *n* = 20, has the highest PRE, coming in at 105.42. In a similar vein, for *n* = 50 and 80, $T_{KM_{st}}$ additionally displays 105.98 and 105.99 as dominant PRE values, respectively. Similarly, the suggested generalized mixture estimator exhibits the highest PREs in dataset II. This implies that when compared to comparative estimators, the suggested estimator performs remarkably well and efficiently. Furthermore, it is clear that the PREs rise in tandem with larger sample sizes. In Fig 3, the suggested estimator’s performance is further illustrated graphically.

**Table 12:** Estimated sample means and proposed generalized mixture estimators’ percent relative efficiencies (PREs) with comparative estimators’ PREs for data set I.

Estimators	Estimated Sample Means			Data I		
	*n* = 20	*n* = 50	*n* = 80	*n* = 20	*n* = 50	*n* = 80
${\hat{\mu }}_{y,StRS}$	1.06	1.19	1.30	100	100	100
${\hat{\mu }}_{yKC_{\text{1}},StRS}$	1.61	1.64	1.69	101.35	101.84	102.12
${\hat{\mu }}_{yKC_{\text{2}},StRS}$	1.40	1.53	1.66	101.41	101.65	101.98
${\hat{\mu }}_{yKC_{3},StRS}$	1.51	1.59	1.65	101.86	101.9	101.99
${\hat{\mu }}_{yKC_{4},StRS}$	1.24	1.45	1.61	101.46	101.6	101.87
${\hat{\mu }}_{yZ_{1},StRS}$	1.11	1.40	1.59	101.37	101.62	101.89
${\hat{\mu }}_{yZ_{2},StRS}$	1.16	1.23	1.38	101.45	101.73	101.98
${\hat{\mu }}_{yZ_{3},StRS}$	1.20	1.39	1.41	101.14	101.38	101.88
${\hat{\mu }}_{yZ_{4},StRS}$	1.12	1.21	1.46	102.99	103.21	103.76
${\hat{\mu }}_{yZ_{5},StRS}$	1.08	1.30	1.43	101.82	101.91	101.98
$T_{KM_{st}}$	1.67	1.71	1.78	**105.42**	**105.98**	**105.99**

**Table 13:** Estimated sample means and proposed generalized mixture estimators’ percent relative efficiencies (PREs) with comparative estimators’ PREs for data set II.

Estimators	Estimated Sample Means			Data II		
	*n* = 20	*n* = 50	*n* = 80	*n* = 20	*n* = 50	*n* = 80
${\hat{\mu }}_{y,StRS}$	635.43	636.44	637.19	100	100	100
${\hat{\mu }}_{yKC_{\text{1}},StRS}$	635.02	636.87	637.06	101.12	101.37	101.67
${\hat{\mu }}_{yKC_{2},StRS}$	636.32	636.52	637.13	101.25	101.41	101.74
${\hat{\mu }}_{yKC_{3},StRS}$	635.52	636.64	637.72	101.45	101.5	101.87
${\hat{\mu }}_{yKC_{4},StRS}$	635.20	636.17	637.12	101.1	101.34	101.79
${\hat{\mu }}_{yZ_{1},StRS}$	634.67	635.98	636.18	101.23	101.62	101.81
${\hat{\mu }}_{yZ_{2},StRS}$	633.42	636.70	637.03	101.32	101.41	101.52
${\hat{\mu }}_{yZ_{3},StRS}$	634.67	635.17	637.61	101.14	101.42	101.86
${\hat{\mu }}_{yZ_{4},StRS}$	633.26	635.11	637.87	101.87	101.89	101.92
${\hat{\mu }}_{yZ_{5},StRS}$	632.56	633.30	636.38	101.78	101.81	101.88
$T_{KM_{st}}$	636.93	637.35	638.99	**104.98**	**105.12**	**105.34**

## Conclusion and recommendations

In different scenarios, using auxiliary information is a very useful strategy to improve the estimator’s efficiency. For this purpose, we have used auxiliary variables and attributes simultaneously. In this study, we introduce a generalized family of mixture estimators under Stratified Random Sampling (StRS), inspired by the work of Zaman [25], aimed at enhancing efficiency across symmetric and asymmetric distributions for estimating finite population means. Additionally, we analyze the estimator’s behavior across various sample sizes regarding its convergence to the Normal distribution. Our findings indicate that the proposed mixture estimator adheres to the Normal distribution for sample sizes greater than or equal to 35. Furthermore, we derive the Mean Squared Error (MSE) expressions for the proposed estimator, supported by simulation results. Notably, our proposed class of estimators outperforms competing estimators in terms of Percent Relative Efficiency (PRE). Through simulation studies and real-life applications in the health and finance sectors, we demonstrate that our proposed estimator consistently delivers superior results compared to competitors across Normal, Uniform, Weibull, and Gamma distributions. Ultimately, we conclude that the efficiency of the suggested estimator holds both theoretically and in practical settings. Moreover, we suggest extending the scope of this study to include other types of estimators, such as ratio, product, power, difference, exponential, and regression estimators, under stratified random sampling.

## Supporting information

S1 Appendix (DOCX)
