# Supplementary material for: Exploring mixture estimators in stratified random sampling
Source: PLoS One. 2024 Sep 17;19(9):e0307607. doi: 10.1371/journal.pone.0307607 (PMC11407636; doi:10.1371/journal.pone.0307607)
Supplement: S1 Appendix — (DOCX) [file pone.0307607.s001.docx]

**Appendix**

**A.1. Definition of the estimator** $\boldsymbol{T}_{\boldsymbol{K}\boldsymbol{M}_{\boldsymbol{h}}}$

$$T_{KMst}=\sum_{h=1}^{k} \frac{N_{h}}{N}\left[ aT_{p_{h}}+\left( 1-a \right)T_{x_{h}} \right],$$

where

$$T_{P_{h}}=\overline{y}_{h}P_{U},T_{x_{h}}=\overline{y}_{h}\overline{X}_{U},$$

$$\left. \begin{matrix} P_{U}=\left( P_{h}K_{1}+K_{2} \right)and\overline{X}_{U}=\left( \overline{X}_{h}K_{3}+K_{4} \right) \\ p_{U}=\left( \hat{P}_{h}K_{1}+K_{2} \right)and\overline{x}_{U}=\left( {\hat{\overline{X}}}_{h}K_{3}+K_{4} \right) \end{matrix} \right\},$$

where a takes value either 0 and 1 and $K\in\mathbb{R}$, we may rewrite it as,

$$T_{KMst}=\sum_{h=1}^{k} \frac{N_{h}}{N}\left[ a\left\{ \overline{y}_{h}\frac{P_{U}}{\hat{P}_{U}} \right\}+\left( 1-a \right)\left\{ \overline{y}_{h}\frac{\overline{X}_{U}}{{\hat{\overline{X}}}_{h}} \right\} \right].$$

Where for the $h^{th}$stratum, let $T_{KM_{h}}$be a mixture estimator of the population mean given by:

$$T_{KM_{h}}=\left[ a\left\{ \overline{y}_{h}\frac{P_{U}}{\hat{P}_{U}} \right\}+\left( 1-a \right)\left\{ \overline{y}_{h}\frac{\overline{X}_{U}}{{\hat{\overline{X}}}_{U}} \right\} \right].$$

**A.2. Derivation of** $\boldsymbol{Bias}\left( \boldsymbol{T}_{\boldsymbol{K}\boldsymbol{M}_{\boldsymbol{h}}} \right)$**and**$\boldsymbol{MSE}\left( \boldsymbol{T}_{\boldsymbol{K}\boldsymbol{M}_{\boldsymbol{h}}} \right)$

We may rewrite $T_{KM_{h}}$ as,

$$T_{KM_{h}}=\overline{y}_{h}\left[ a\left\{ \frac{P_{U_{h}}}{\hat{P}_{Uh}} \right\}+\left( 1-a \right)\left\{ \frac{\overline{X}_{U_{h}}}{{\hat{\overline{X}}}_{Uh}} \right\} \right],$$

or

$$T_{KM_{h}}=\overline{y}_{h}\left[ a\left\{ \frac{P_{U_{h}}}{\hat{P}_{Uh}} \right\}+\left( 1-a \right)\left\{ \frac{\overline{X}_{U_{h}}}{{\hat{\overline{X}}}_{Uh}} \right\} \right],$$

$$T_{KM_{h}}=\overline{y}_{h}\left[ a\left\{ \frac{P_{U_{h}}}{\hat{P}_{Uh}} \right\}+\left( 1-a \right)\left\{ \frac{\overline{X}_{U_{h}}}{{\hat{\overline{X}}}_{Uh}} \right\} \right],$$

By using notations, the bias of $T_{KM_{h}}$is derived as follows, and we get,

$$T_{KM_{h}}=\overline{Y}_{h}\left( 1+e_{0} \right)\left[ a\left\{ \frac{P_{U_{h}}}{P_{U_{h}}\left( 1+e_{3} \right)} \right\}+\left( 1-a \right)\left\{ \frac{\overline{X}_{U_{h}}}{\overline{X}_{U_{h}}\left( 1+e_{4} \right)} \right\} \right],$$

$$T_{KM_{h}}=\overline{Y}_{h}\left( 1+e_{0} \right)\left[ a\left( 1+e_{3} \right)^{-1}+\left( 1-a \right)\left( 1+e_{4} \right)^{-1} \right],$$

$$T_{KM_{h}}=\overline{Y}_{h}\left( 1+e_{0} \right)\left[ a\left( 1-e_{3}+e_{3}^{2}...... \right)+\left( 1-a \right)\left( 1-e_{4}+e_{4}^{2}...... \right) \right],$$

Using the Taylor series and neglecting the high-order terms we get,

$$T_{KM_{h}}=\overline{Y}_{h}\left( 1+e_{0} \right)\left[ a\left( 1-e_{3}+e_{3}^{2} \right)+\left( 1-a \right)\left( 1-e_{4}+e_{4}^{2} \right) \right],$$

$$T_{KM_{h}}=\overline{Y}_{h}\left[ a\left( 1-e_{3}+e_{3}^{2}+e_{0}-e_{0}e_{3} \right)+\left( 1-a \right)\left( 1-e_{4}+e_{4}^{2}+e_{0}-e_{0}e_{4} \right) \right],$$

$T_{KM_{h}}=\overline{Y}_{h}\left[ \left( 1-e_{4}+e_{4}^{2}+e_{0}-e_{0}e_{4} \right)+a\left( 1-e_{3}+e_{3}^{2}+e_{0}-e_{0}e_{3}-1+e_{4}-e_{4}^{2}+e_{0}e_{4}-e_{0} \right) \right],$ $T_{KM_{h}}=\overline{Y}_{h}\left[ \left( 1-e_{4}+e_{0}-e_{0}e_{4}+e_{4}^{2} \right)+a\left( -e_{3}+e_{3}^{2}-e_{0}e_{3}+e_{4}-e_{4}^{2}+e_{0}e_{4} \right) \right],$

$$T_{KM_{h}}-\overline{Y}_{h}=\overline{Y}_{h}\left[ \left( e_{0}-e_{4}-e_{0}e_{4}+e_{4}^{2} \right)+a\left( -e_{3}+e_{3}^{2}-e_{0}e_{4}+e_{4}-e_{4}^{2}+e_{0}e_{4} \right) \right],$$

Taking expectations on both sides

$$E\left[ T_{KM_{h}}-\overline{Y}_{h} \right]=\overline{Y}_{h}E\left[ \left( e_{0}-e_{4}-e_{0}e_{4}+e_{4}^{2} \right)+a\left( -e_{3}+e_{3}^{2}-e_{0}e_{4}+e_{4}-e_{4}^{2}+e_{0}e_{4} \right) \right],$$

$$Bias\left[ T_{KM_{h}} \right]=\overline{Y}_{h}E\left[ \left( e_{0}-e_{4}-e_{0}e_{4}+e_{4}^{2} \right)+a\left( -e_{3}+e_{3}^{2}-e_{0}e_{4}+e_{4}-e_{4}^{2}+e_{0}e_{4} \right) \right],$$

Using notations, we get,

$$Bias\left[ T_{KM_{h}} \right]=\overline{Y}_{h}\left[ \left( 0-0-\frac{\lambda_{h}S_{yx_{h}}}{\overline{Y}_{h}.\overline{X}_{U_{h}}}+\frac{\lambda_{h}S_{x_{h}}^{2}}{\overline{X}_{U_{h}}^{2}} \right)+a\left( \frac{\lambda_{h}S_{p_{h}}^{2}}{P_{U_{h}}^{2}}-\frac{\lambda_{h}S_{yp_{h}}}{\overline{Y}_{h}.P_{U_{h}}}-\frac{\lambda_{h}S_{x_{h}}^{2}}{\overline{X}_{U_{h}}^{2}}+\frac{\lambda_{h}S_{yx_{h}}}{\overline{Y}_{h}.\overline{X}_{U_{h}}} \right) \right],$$

$$Bias\left[ T_{KM_{h}} \right]=\overline{Y}_{h}\left[ \left( \frac{\lambda_{h}S_{x_{h}}^{2}}{\overline{X}_{U_{h}}^{2}}-\frac{\lambda_{h}S_{yx_{h}}}{\overline{Y}_{h}.\overline{X}_{U_{h}}} \right)+a\left( \frac{\lambda_{h}S_{p_{h}}^{2}}{P_{U_{h}}^{2}}-\frac{\lambda_{h}S_{yp_{h}}}{\overline{Y}_{h}.P_{U_{h}}}-\frac{\lambda_{h}S_{x_{h}}^{2}}{\overline{X}_{U_{h}}^{2}}+\frac{\lambda_{h}S_{yx_{h}}}{\overline{Y}_{h}.\overline{X}_{U_{h}}} \right) \right],$$

$$Bias\left[ T_{KM_{h}} \right]=\overline{Y}_{h}\left[ \lambda_{h}\left( \frac{S_{x_{h}}^{2}}{\overline{X}_{U_{h}}^{2}}-\frac{S_{yx_{h}}}{\overline{Y}_{h}.\overline{X}_{U_{h}}} \right)+a\lambda_{h}\left( \frac{S_{p_{h}}^{2}}{P_{U_{h}}^{2}}-\frac{S_{yp_{h}}}{\overline{Y}_{h}.P_{U_{h}}}-\frac{S_{x_{h}}^{2}}{\overline{X}_{U_{h}}^{2}}+\frac{S_{yx_{h}}}{\overline{Y}_{h}.\overline{X}_{U_{h}}} \right) \right],$$

$$Bias\left[ T_{KM_{h}} \right]=\lambda_{h}\left( \frac{\overline{Y}_{h}S_{x_{h}}^{2}}{\overline{X}_{U_{h}}^{2}}-\frac{S_{yx_{h}}}{\overline{X}_{U_{h}}} \right)+a\lambda_{h}\left( \frac{\overline{Y}_{h}S_{p_{h}}^{2}}{P_{U_{h}}^{2}}-\frac{S_{yp_{h}}}{P_{U_{h}}}-\frac{\overline{Y}_{h}S_{x_{h}}^{2}}{\overline{X}_{U_{h}}^{2}}+\frac{S_{yx_{h}}}{\overline{X}_{U_{h}}} \right).$$

Hence, the Bias expression of $T_{KM_{st}}$is obtained as follows:

$$Bias\left( T_{KMst} \right)=\sum_{h=1}^{k} \frac{N_{h}^{2}}{N^{2}}Bias\left[ T_{KM_{h}} \right]$$

$$Bias\left[ T_{KM_{st}} \right]=\sum_{h=1}^{k} \frac{N_{h}^{2}}{N^{2}}\left( \frac{\overline{Y}_{h}S_{x_{h}}^{2}}{\overline{X}_{U_{h}}^{2}}-\frac{S_{y_{h}x_{h}}}{\overline{X}_{U_{h}}} \right)+a\sum_{h=1}^{k} \frac{N_{h}^{2}}{N^{2}}\left( \frac{\overline{Y}_{h}S_{p_{h}}^{2}}{P_{U_{h}}^{2}}-\frac{S_{y_{h}p_{h}}}{P_{U_{h}}}-\frac{\overline{Y}_{h}S_{x_{h}}^{2}}{\overline{X}_{U_{h}}^{2}}+\frac{S_{y_{h}x_{h}}}{\overline{X}_{U_{h}}} \right).$$

**Derivation of** $\boldsymbol{MSE}\left[ \boldsymbol{T}_{\boldsymbol{K}\boldsymbol{M}_{\boldsymbol{h}}} \right]$ **is as follows:**

Taking expectation and square on both sides

$$E\left[ T_{KM_{h}}-\overline{Y}_{h} \right]^{2}=\left[ \overline{Y}_{h}E\left[ \left( e_{0}-e_{4}-e_{0}e_{4}+e_{4}^{2} \right)+a\left( -e_{3}+e_{3}^{2}-e_{0}e_{4}+e_{4}-e_{4}^{2}+e_{0}e_{4} \right) \right] \right]^{2},$$

$$MSE\left[ T_{KM_{h}} \right]=\overline{Y}_{h}^{2}E\left[ \left( e_{0}^{2}+e_{4}^{2}-2e_{0}e_{4} \right)+a^{2}\left( e_{3}^{2}+e_{4}^{2}-e_{3}e_{4} \right)+2a\left( e_{0}e_{4}-e_{0}e_{3}+e_{3}e_{4}-e_{4}^{2} \right) \right],$$

Using the notations, we get,

$$MSE\left( T_{KM_{h}} \right)=\overline{Y}_{h}^{2}\left[ \begin{aligned} &\left( \frac{\lambda_{h}S_{y_{h}}^{2}}{\overline{Y}_{h}^{2}}+\frac{\lambda_{h}S_{x_{h}}^{2}}{\overline{X}_{U_{h}}^{2}}-\frac{2\lambda_{h}S_{yx_{h}}}{\overline{Y}_{h}.\overline{X}_{U_{h}}} \right)+a^{2}\left( \frac{\lambda_{h}S_{p_{h}}^{2}}{P_{U_{h}}^{2}}+\frac{\lambda_{h}S_{x_{h}}^{2}}{\overline{X}_{U_{h}}^{2}}-\frac{2\lambda_{h}S_{xp_{h}}}{\overline{X}_{U_{h}}.P_{U_{h}}} \right) \\ &+2a\left( \frac{\lambda_{h}S_{yx_{h}}}{\overline{Y}_{h}.\overline{X}_{U_{h}}}-\frac{\lambda_{h}S_{yp_{h}}}{\overline{Y}_{h}.P_{U_{h}}}+\frac{\lambda_{h}S_{xp_{h}}}{\overline{X}_{U_{h}}.P_{U_{h}}}-\frac{\lambda_{h}S_{x_{h}}^{2}}{\overline{X}_{U_{h}}^{2}} \right) \end{aligned} \right],$$

$$MSE\left( T_{KM_{h}} \right)=\left[ \begin{aligned} &\overline{Y}_{h}^{2}\left( \frac{\lambda_{h}S_{y_{h}}^{2}}{\overline{Y}_{h}^{2}}+\frac{\lambda_{h}S_{x_{h}}^{2}}{\overline{X}_{U_{h}}^{2}}-\frac{2\lambda_{h}S_{yx_{h}}}{\overline{Y}_{h}.\overline{X}_{U_{h}}} \right)+\overline{Y}_{h}^{2}a^{2}\left( \frac{\lambda_{h}S_{p_{h}}^{2}}{P_{U_{h}}^{2}}+\frac{\lambda_{h}S_{x_{h}}^{2}}{\overline{X}_{U_{h}}^{2}}-\frac{2\lambda_{h}S_{xp_{h}}}{\overline{X}_{U_{h}}.P_{U_{h}}} \right) \\ &+\overline{Y}_{h}^{2}2a\left( \frac{\lambda_{h}S_{yx_{h}}}{\overline{Y}_{h}.\overline{X}_{U_{h}}}-\frac{\lambda_{h}S_{yp_{h}}}{\overline{Y}_{h}.P_{U_{h}}}+\frac{\lambda_{h}S_{xp_{h}}}{\overline{X}_{U_{h}}.P_{U_{h}}}-\frac{\lambda_{h}S_{x_{h}}^{2}}{\overline{X}_{U_{h}}^{2}} \right) \end{aligned} \right],$$

$$MSE\left( T_{KM_{h}} \right)=\left[ \begin{aligned} &\overline{Y}_{h}^{2}\lambda_{h}\left( \frac{S_{y_{h}}^{2}}{\overline{Y}_{h}^{2}}+\frac{S_{x_{h}}^{2}}{\overline{X}_{U_{h}}^{2}}-\frac{2S_{yx_{h}}}{\overline{Y}_{h}.\overline{X}_{U_{h}}} \right)+\overline{Y}_{h}^{2}a^{2}\lambda_{h}\left( \frac{S_{p_{h}}^{2}}{P_{U_{h}}^{2}}+\frac{S_{x_{h}}^{2}}{\overline{X}_{U_{h}}^{2}}-\frac{2S_{xp_{h}}}{\overline{X}_{U_{h}}.P_{U_{h}}} \right) \\ &+\overline{Y}_{h}^{2}2a\lambda_{h}\left( \frac{S_{yx_{h}}}{\overline{Y}_{h}.\overline{X}_{U_{h}}}-\frac{S_{yp_{h}}}{\overline{Y}_{h}.P_{U_{h}}}+\frac{S_{xp_{h}}}{\overline{X}_{U_{h}}.P_{U_{h}}}-\frac{S_{x_{h}}^{2}}{\overline{X}_{U_{h}}^{2}} \right) \end{aligned} \right],$$

$$MSE\left( T_{KM_{h}} \right)=\left[ \begin{aligned} &\lambda_{h}\left( S_{y_{h}}^{2}+\frac{\overline{Y}_{h}^{2}S_{x_{h}}^{2}}{\overline{X}_{U_{h}}^{2}}-\frac{2\overline{Y}_{h}S_{yx_{h}}}{\overline{X}_{U_{h}}} \right)+a^{2}\lambda_{h}\left( \frac{\overline{Y}_{h}^{2}S_{p}^{2}}{P_{U_{h}}^{2}}+\frac{\overline{Y}_{h}^{2}S_{x}^{2}}{\overline{X}_{U_{h}}^{2}}-\frac{2\overline{Y}_{h}^{2}S_{xp}}{\overline{X}_{U_{h}}.P_{U_{h}}} \right) \\ &+2a\lambda_{h}\left( \frac{\overline{Y}_{h}S_{yx_{h}}}{\overline{X}_{U_{h}}}-\frac{\overline{Y}_{h}S_{yp_{h}}}{P_{U_{h}}}+\frac{\overline{Y}_{h}^{2}S_{xp_{h}}}{\overline{X}_{U_{h}}.P_{U_{h}}}-\frac{\overline{Y}_{h}^{2}S_{x_{h}}^{2}}{\overline{X}_{U_{h}}^{2}} \right) \end{aligned} \right],$$

Partially differentiating $MSE\left( T_{KM_{h}} \right)$ and with respect to "$a$" and equating the result to zero gives the maximum value of "$a$". We obtain the equations for $a$:

$$\frac{\partial MSE\left( T_{KM_{h}} \right)}{\partial a}=0$$

gives,

$$0+2a^{*}\lambda_{h}\left[ \frac{\overline{Y}_{h}^{2}S_{p_{h}}^{2}}{P_{U_{h}}^{2}}+\frac{\overline{Y}_{h}^{2}S_{x}^{2}}{\overline{X}_{U_{h}}^{2}}+\frac{2\overline{Y}_{h}^{2}S_{xp_{h}}}{\overline{X}_{U_{h}}.P_{U_{h}}} \right]+2\lambda_{h}\left[ \frac{\overline{Y}_{h}S_{yx_{h}}}{\overline{X}_{U_{h}}}- \right.\frac{\overline{Y}_{h}S_{yp_{h}}}{P_{U_{h}}}+\left. \frac{\overline{Y}_{h}^{2}S_{xp}}{\overline{X}_{U_{h}}.P_{U_{h}}}-\frac{\overline{Y}_{h}^{2}S_{x}^{2}}{\overline{X}_{U_{h}}^{2}} \right]=0,$$

or

$$a\frac{\overline{Y}_{h}^{2}S_{p_{h}}^{2}\overline{X}_{U_{h}}^{2}+\overline{Y}_{h}^{2}S_{x_{h}}^{2}P_{U_{h}}^{2}+2\overline{Y}_{h}^{2}S_{xp_{h}}\overline{X}_{U_{h}}.P_{U_{h}}}{P_{U_{h}}^{2}\overline{X}_{U_{h}}^{2}}=-\frac{\overline{Y}_{h}S_{yx_{h}}\overline{X}_{U_{h}}.P_{U_{h}}^{2}+\overline{Y}_{h}S_{yp}P_{U_{h}}\overline{X}_{U_{h}}^{2}\overline{Y}_{h}^{2}S_{xp_{h}}\overline{X}_{U_{h}}.P_{U_{h}}+\overline{Y}_{h}^{2}S_{x_{h}}^{2}P_{U_{h}}^{2}}{P_{U_{h}}^{2}\overline{X}_{U_{h}}^{2}},$$

or

$$a=-\frac{\overline{Y}_{h}S_{yx_{h}}\overline{X}_{U_{h}}.P_{U_{h}}^{2}+\overline{Y}_{h}S_{yp_{h}}P_{U_{h}}\overline{X}_{U_{h}}^{2}\overline{Y}_{h}^{2}S_{xp_{h}}\overline{X}_{U_{h}}.P_{U_{h}}+\overline{Y}_{h}^{2}S_{x_{h}}^{2}P_{U_{h}}^{2}}{\overline{Y}_{h}^{2}S_{p_{h}}^{2}\overline{X}_{U_{h}}^{2}+\overline{Y}_{h}^{2}S_{x_{h}}^{2}P_{U_{h}}^{2}+2\overline{Y}_{h}^{2}S_{xp_{h}}\overline{X}_{U_{h}}.P_{U_{h}}},$$

or

$$a=\frac{\frac{1}{\overline{Y}_{h}}\left[ -S_{yx_{h}}\overline{X}_{U_{h}}.P_{U_{h}}^{2}+S_{yp_{h}}P_{U_{h}}\overline{X}_{U_{h}}^{2} \right]-S_{xp_{h}}\overline{X}_{U_{h}}.P_{U_{h}}+S_{x_{h}}^{2}P_{U_{h}}^{2}}{S_{p_{h}}^{2}\overline{X}_{U_{h}}^{2}+S_{x}^{2}P_{U_{h}}^{2}+2S_{xp_{h}}\overline{X}_{U_{h}}.P_{U_{h}}},$$

or

$$a=\frac{\frac{\overline{X}_{U_{h}}.P_{U_{h}}}{\overline{Y}_{h}}\left[ -S_{yx_{h}}P_{U_{h}}+S_{yp_{h}}\overline{X}_{U_{h}} \right]+P_{U_{h}}\left[ -S_{xp_{h}}\overline{X}_{U_{h}}+S_{x_{h}}^{2}P_{U_{h}} \right]}{S_{p_{h}}^{2}\overline{X}_{U_{h}}^{2}+S_{x}^{2}P_{U_{h}}^{2}+2S_{xp_{h}}\overline{X}_{U_{h}}.P_{U_{h}}},$$

or

$$a=\frac{\overline{X}_{U_{h}}.P_{U_{h}}\left[ -S_{yx_{h}}P_{U_{h}}+S_{yp_{h}}\overline{X}_{U_{h}} \right]+\overline{Y}_{h}P_{U_{h}}\left[ -S_{xp_{h}}\overline{X}_{U_{h}}+S_{x_{h}}^{2}P_{U_{h}} \right]}{\overline{Y}_{h}\left[ S_{p_{h}}^{2}\overline{X}_{U_{h}}^{2}+S_{x_{h}}^{2}P_{U_{h}}^{2}+2S_{xp_{h}}\overline{X}_{U_{h}}.P_{U_{h}} \right]}.$$

Putting the value of $a$ in $MSE\left( T_{KM_{h}} \right)$ we obtain:

$=\lambda_{h}\left( S_{y_{h}}^{2}+\frac{\overline{Y}_{h}^{2}S_{x_{h}}^{2}}{\overline{X}_{U_{h}}^{2}}-\frac{2\overline{Y}_{h}S_{yx_{h}}}{\overline{X}_{U_{h}}} \right)+\lambda_{h}\left( \begin{aligned} &\frac{\overline{X}_{U_{h}}^{2}P_{U_{h}}^{2}\left\{ \left[ -S_{yx_{h}}P_{U_{h}}+S_{yp_{h}}\overline{X}_{U_{h}} \right]+\frac{\overline{Y}_{h}}{\overline{X}_{U_{h}}}\left[ -S_{xp_{h}}\overline{X}_{U_{h}}+S_{x_{h}}^{2}P_{U_{h}} \right] \right\}^{2}}{\frac{\overline{Y}_{h}^{2}}{\overline{Y}_{h}^{4}}\left[ \overline{Y}_{h}^{2}S_{p_{h}}^{2}\overline{X}_{U_{h}}^{2}+S_{x_{h}}^{2}P_{U_{h}}^{2}+2S_{xp_{h}}\overline{X}_{U_{h}}.P_{U_{h}} \right]^{2}}\times\\ &\frac{\left[ \overline{Y}_{h}^{2}S_{p}^{2}\overline{X}_{U_{h}}^{2}+\overline{Y}_{h}^{2}S_{x}^{2}P_{U_{h}}^{2}-2\overline{Y}_{h}^{2}S_{xp}\overline{X}_{U_{h}}.P_{U_{h}} \right]^{2}}{P_{U_{h}}^{2}\overline{X}_{U_{h}}^{2}}. \end{aligned} \right)$

$$+2\lambda_{h}\left( \begin{aligned} &\overline{Y}_{h}\frac{\overline{X}_{U_{h}}.P_{U_{h}}\left\{ \left[ -S_{yx_{h}}P_{U_{h}}+S_{yp_{h}}\overline{X}_{U_{h}} \right]+\frac{\overline{Y}_{h}}{\overline{X}_{U_{h}}}\left[ -S_{xp_{h}}\overline{X}_{U_{h}}+S_{x_{h}}^{2}P_{U_{h}} \right] \right\}}{\overline{Y}_{h}\left[ S_{p_{h}}^{2}\overline{X}_{U_{h}}^{2}+S_{x}^{2}P_{U_{h}}^{2}+2S_{xp_{h}}\overline{X}_{U_{h}}.P_{U_{h}} \right]}\times\\ &\frac{S_{yx_{h}}\overline{X}_{U_{h}}.P_{U_{h}}-S_{yp_{h}}\overline{X}_{U_{h}}+\overline{Y}_{h}S_{xp_{h}}\overline{X}_{U_{h}}-\overline{Y}_{h}S_{x_{h}}^{2}P_{U_{h}}}{\overline{X}_{U_{h}}^{2}P_{U_{h}}} \end{aligned} \right),$$

$=\left[ \begin{aligned} &\lambda_{h}\left( S_{y_{h}}^{2}+\frac{\overline{Y}_{h}^{2}S_{x}^{2}}{\overline{X}_{U_{h}}^{2}}-\frac{2\overline{Y}_{h}S_{yx_{h}}}{\overline{X}_{U_{h}}} \right)+ \\ &\lambda_{h}\left( \frac{\left[ -S_{yx_{h}}P_{U_{h}}+S_{yp_{h}}\overline{X}_{U_{h}} \right]^{2}+\frac{\overline{Y}_{h}^{2}}{\overline{X}_{U_{h}}^{2}}\left[ -S_{xp_{h}}\overline{X}_{U_{h}}+S_{x_{h}}^{2}P_{U_{h}} \right]^{2}+\frac{2\overline{Y}_{h}}{\overline{X}_{U_{h}}}\left[ -S_{yx_{h}}P_{U_{h}}+S_{yp_{h}}\overline{X}_{U_{h}} \right]\left[ -S_{xp_{h}}\overline{X}_{U_{h}}+S_{x_{h}}^{2}P_{U_{h}} \right]^{2}}{S_{p_{h}}^{2}\overline{X}_{U_{h}}^{2}+S_{x_{h}}^{2}P_{U_{h}}^{2}+2S_{xp}\overline{X}_{U_{h}}.P_{U_{h}}} \right) \\ &+2\lambda_{h}\left( \frac{\left\{ \left[ -S_{yx_{h}}P_{U_{h}}+S_{yp_{h}}\overline{X}_{U_{h}} \right]+\frac{\overline{Y}_{h}}{\overline{X}_{U_{h}}}\left[ -S_{xp_{h}}\overline{X}_{U_{h}}+S_{x_{h}}^{2}P_{U_{h}} \right] \right\}\times\left\{ \left. \left[ -S_{yx_{h}}P_{U_{h}}+S_{yp_{h}}\overline{X}_{U_{h}} \right. \right]-\frac{\overline{Y}_{h}}{\overline{X}_{U_{h}}}\left[ -S_{xp_{h}}\overline{X}_{U_{h}}-\overline{Y}_{h}S_{x_{h}}^{2}P_{U_{h}} \right] \right\}}{S_{p_{h}}^{2}\overline{X}_{U_{h}}^{2}+S_{x_{h}}^{2}P_{U_{h}}^{2}+2S_{xp_{h}}\overline{X}_{U_{h}}.P_{U_{h}}}. \right) \end{aligned} \right],$

$$MSE\left( T_{KM_{h}} \right)=\left[ \begin{aligned} &\lambda_{h}\left( S_{y_{h}}^{2}+\frac{\overline{Y}_{h}^{2}S_{x_{h}}^{2}}{\overline{X}_{U_{h}}^{2}}-\frac{2\overline{Y}_{h}S_{yx_{h}}}{\overline{X}_{U_{h}}} \right)-\lambda_{h}\frac{\left[ -S_{yx_{h}}P_{U_{h}}+S_{yp_{h}}\overline{X}_{U_{h}} \right]+\frac{\overline{Y}_{h}}{\overline{X}_{U_{h}}}\left[ -S_{xp_{h}}\overline{X}_{U_{h}}+S_{x_{h}}^{2}P_{U_{h}} \right]^{2}}{S_{p_{h}}^{2}\overline{X}_{U_{h}}^{2}+S_{x_{h}}^{2}P_{U_{h}}^{2}+2S_{xp}\overline{X}_{U_{h}}.P_{U_{h}}} \\ &+2\lambda_{h}\frac{\left\{ \left[ -S_{yx_{h}}P_{U_{h}}+S_{yp_{h}}\overline{X}_{U_{h}} \right]+\frac{\overline{Y}_{h}}{\overline{X}_{U}}\left[ -S_{xp_{h}}\overline{X}_{U_{h}}+S_{x_{h}}^{2}P_{U_{h}} \right] \right\}^{2}}{S_{p_{h}}^{2}\overline{X}_{U_{h}}^{2}+S_{x_{h}}^{2}P_{U_{h}}^{2}+2S_{xp_{h}}\overline{X}_{U_{h}}.P_{U_{h}}} \end{aligned} \right],$$

$$MSE\left( T_{KM_{h}} \right)=\left[ \begin{aligned} &\lambda_{h}\left( S_{y_{h}}^{2}+\frac{\overline{Y}_{h}^{2}S_{x_{h}}^{2}}{\overline{X}_{U_{h}}^{2}}-\frac{2\overline{Y}_{h}S_{yx_{h}}}{\overline{X}_{U_{h}}} \right)+ \\ &\lambda_{h}\frac{\left[ -S_{yx_{h}}P_{U_{h}}+S_{yp_{h}}\overline{X}_{U_{h}} \right]+\frac{\overline{Y}_{h}}{\overline{X}_{U_{h}}}\left[ -S_{xp_{h}}\overline{X}_{U_{h}}+S_{x_{h}}^{2}P_{U_{h}} \right]^{2}}{S_{p_{h}}^{2}\overline{X}_{U_{h}}^{2}+S_{x_{h}}^{2}P_{U_{h}}^{2}+2S_{xp_{h}}\overline{X}_{U_{h}}.P_{U_{h}}} \end{aligned} \right].$$

Hence, the mean square error expression of $T_{KM_{st}}$ is given by,

$$MSE\left( T_{KMst} \right)=\sum_{h=1}^{k} \frac{N_{h}^{2}}{N^{2}}MSE\left( T_{KM_{h}} \right)$$

$$MSE\left( T_{KMst} \right)=\sum_{h=1}^{k} \frac{N_{h}^{2}}{N^{2}}\left( \begin{aligned} &\lambda_{h}\left( S_{y_{h}}^{2}+\frac{\overline{Y}_{h}^{2}S_{x_{h}}^{2}}{\overline{X}_{U_{h}}^{2}}-\frac{2\overline{Y}_{h}S_{yx_{h}}}{\overline{X}_{U_{h}}} \right)+ \\ &\lambda_{h}\frac{\left[ -S_{yx_{h}}P_{U_{h}}+S_{yp_{h}}\overline{X}_{U_{h}} \right]+\frac{\overline{Y}_{h}}{\overline{X}_{U_{h}}}\left[ -S_{xp_{h}}\overline{X}_{U_{h}}+S_{x_{h}}^{2}P_{U_{h}} \right]^{2}}{S_{p_{h}}^{2}\overline{X}_{U_{h}}^{2}+S_{x_{h}}^{2}P_{U_{h}}^{2}+2S_{xp_{h}}\overline{X}_{U_{h}}.P_{U_{h}}} \end{aligned} \right).$$
